# Distinct mechanical properties in homologous spectrin-like repeats of utrophin

**DOI:** 10.1038/s41598-019-41569-4

**Published:** 2019-03-26

**Authors:** Sivaraman Rajaganapathy, Jackie L. McCourt, Sayan Ghosal, Angus Lindsay, Preston M. McCourt, Dawn A. Lowe, James M. Ervasti, Murti V. Salapaka

**Affiliations:** 1 Department of Electrical and Computer Engineering, University of Minnesota - Twin Cities, Minneapolis, MN 55455 USA; 2 Department of Biochemistry, Molecular Biology, and Biophysics, University of Minnesota - Twin Cities, Minneapolis, MN 55455 USA; 3 Department of Rehabilitation Medicine, University of Minnesota - Twin Cities, Minneapolis, MN 55455 USA

## Abstract

Patients with Duchenne muscular dystrophy (DMD) lack the protein dystrophin, which is a critical molecular component of the dystrophin-glycoprotein complex (DGC). Dystrophin is hypothesized to function as a molecular shock absorber that mechanically stabilizes the sarcolemma of striated muscle through interaction with the cortical actin cytoskeleton via its N-terminal half and with the transmembrane protein β-dystroglycan via its C-terminal region. Utrophin is a fetal homologue of dystrophin that can subserve many dystrophin functions and is therefore under active investigation as a dystrophin replacement therapy for DMD. Here, we report the first mechanical characterization of utrophin using atomic force microscopy (AFM). Our data indicate that the mechanical properties of spectrin-like repeats in utrophin are more in line with the PEVK and Ig-like repeats of titin rather than those reported for repeats in spectrin or dystrophin. Moreover, we measured markedly different unfolding characteristics for spectrin repeats within the N-terminal actin-binding half of utrophin compared to those in the C-terminal dystroglycan-binding half, even though they exhibit identical thermal denaturation profiles. Our results demonstrate dramatic differences in the mechanical properties of structurally homologous utrophin constructs and suggest that utrophin may function as a stiff elastic element in series with titin at the myotendinous junction.

## Introduction

Duchenne muscular dystrophy (DMD) is a fatal muscle disease afflicting one in every 4000 boys^1^ caused by mutations in the DMD gene encoding the 427 kDa cytoplasmic protein dystrophin^2^. Dystrophin is predominantly expressed in striated muscle and interacts with a complex of membrane glycoproteins known as the dystrophin-glycoprotein complex (DGC) at the muscle cell membrane, or sarcolemma^3^. Disease-causing mutations in the DMD gene lead to an absence or loss of function of dystrophin, resulting in loss of sarcolemmal integrity and muscle fiber death^4^.

**Figure 1:**
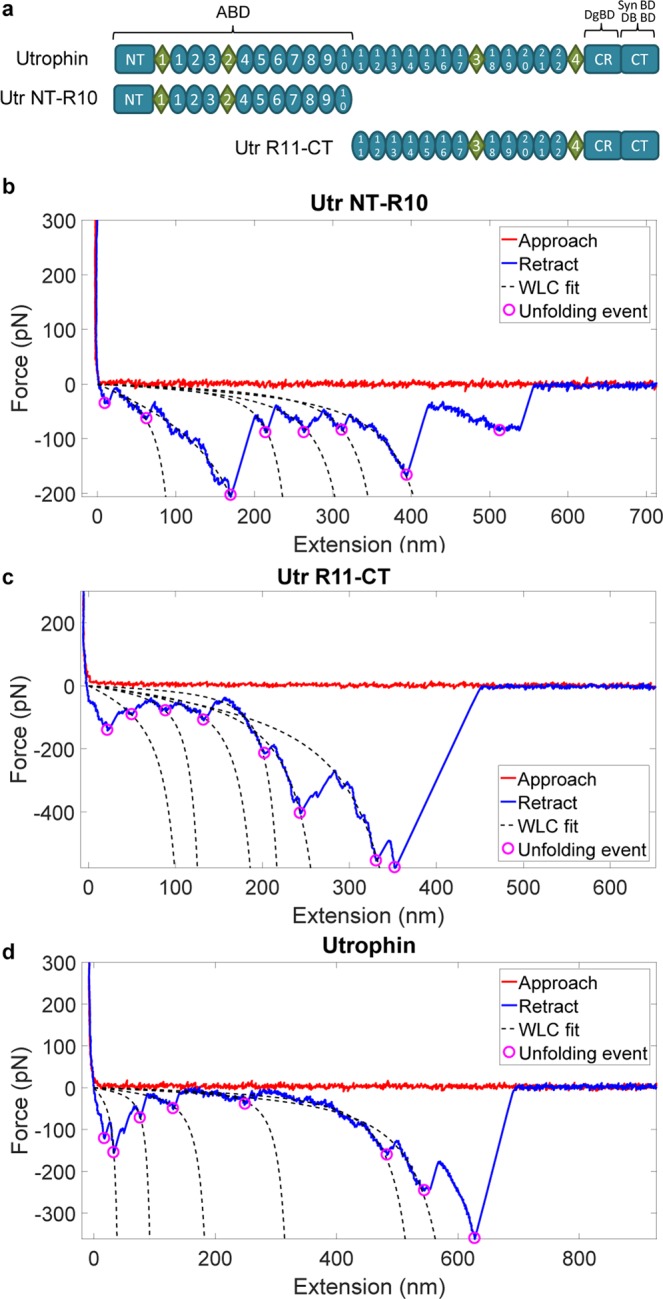
AFM extension characteristics of utrophin terminal constructs. (**a**) Schematic of constructs analyzed by AFM. ABD - actin binding domain; DgBD - dystroglycan binding domain; SynBD - syntrophin binding domain; DBBD - dystrobrevin binding domain; NT - N-terminus; CR - cysteine rich domain; CT - C-terminus; ovals - spectrin-like repeats; diamonds - unstructured “hinge” regions. (**b**–**d**) Force vs extension representative trace curves for Utr NT-R10 (**b**), Utr R11-CT (**c**) and full-length utrophin (**d**). Approach – force on the cantilever as it approaches the substrate; Retract – force on the cantilever as the molecule is extended; Unfolding events – force minima corresponding to domain unfolding. WLC – worm like chain model fit to the force-extension behavior.

Dystrophin is composed of three major functional domains: an N-terminal calponin homology actin binding domain (ABD1), a large central rod domain containing triple helical spectrin-like repeats with a second actin binding domain (ABD2) located within repeats 11–17, and a cysteine-rich C-terminal (CRCT) domain that binds the transmembrane dystroglycan complex and other proteins. It has long been hypothesized that dystrophin acts as a molecular spring or shock absorber to mechanically stabilize the sarcolemma during muscle contraction^5^. Because the homologous protein utrophin can compensate for dystrophin deficiency in the *mdx* mouse model^6^, pharmacologic upregulation of utrophin is under investigation as a therapeutic approach for DMD^7^. Like dystrophin, utrophin forms a homologous utrophin-glycoprotein complex (UGC), but is most abundantly expressed during fetal development and subsequently replaced by dystrophin at the sarcolemma after birth^8^. In adult skeletal muscle, utrophin localizes to the neuromuscular and myotendinous junctions^9^. While many biochemical investigations of dystrophin and utrophin are available, there are only three reports where mechanical characteristics of small recombinant fragments of dystrophin are studied^10–12^; there are no studies on the mechanical characterization of larger fragments and full length utrophin. Here, we report atomic force microscopy analysis of single protein molecules representing the N-terminal actin-binding half (Utr NT-R10), the C-terminal dystroglycan-binding half (Utr R11-CT), and full-length utrophin (Fig. 1a).

## Results

**Figure 2:**
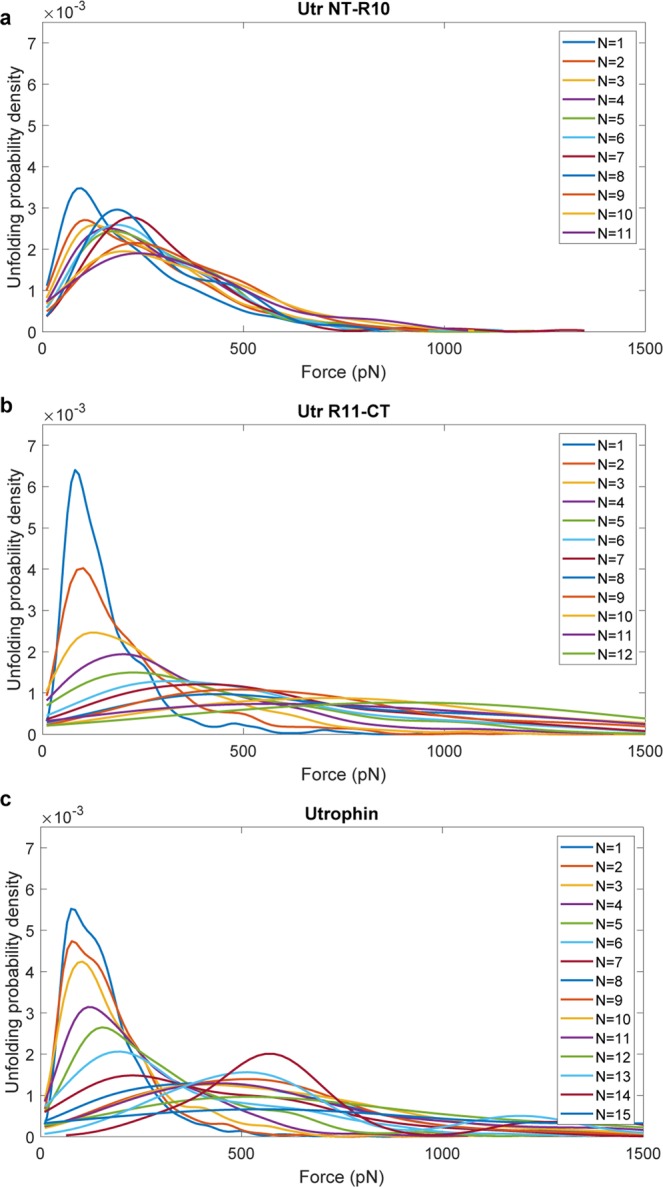
Unfolding force distributions for utrophin terminal constructs reveal markedly different mechanical behaviors. (**a**–**c**) Plots of the probability distribution of unfolding force vs the unfolding force for Utr NT-R10 (**a**), Utr-R11-CT (**b**) and full-length utrophin (**c**) collected from 3 batches of 300–500 successful pulls per batch. Here, ‘N’ represents the unfolding event count. For example, the distribution corresponding to N = 4 represents the distribution of the 4th unfolding event.

Previous atomic force microscopy (AFM) studies have defined the mechanical properties of titin as a “stiff’ spring^13,14^ and spectrin as a “soft” spring^15–17^. In such experiments, the mechanical extensibility of single protein molecules is measured and the unfolding forces of individual domains upon extension are collected over a large number of experiments to obtain statistical properties of unfolding behaviors. We first obtained AFM data for a recombinant titin reference protein (Supplementary Fig. S1) and spectrin (Supplementary Fig. S2) that corroborate previously published values^14,15^. AFM data for Utr NT-R10, Utr R11-CT, and utrophin, all showed characteristic saw-tooth patterns of individual domain unfolding (Fig. 1b–d). From representative force versus extension curves and from the force vs unfolding event statistics (Supplementary Fig. S3 and Fig. 2), we observe that the unfolding forces for utrophin proteins was significantly larger than the unfolding forces reported for spectrin and for fragments of dystrophin (for example, 90–130 pN in Supplementary Fig. S4 versus < 50 pN^10–12^).

To rule out the possibility of pulling multiple proteins, we adjusted the protein concentrations used for pulling experiments. When using higher concentrations, a large fraction of the cases were observed where the number of unfolding events exceeded the number of available domains in a single molecule, suggesting unfolding of multiple proteins is being observed (Supplementary Fig. S5). The protein concentrations used in our experiments were lowered until such observations were absent. The concentrations were further reduced to ensure that the probability of successful pulls were in the 5–25% range, a success percentage used in previous studies to greatly reduce the probability of pulling multiple proteins^18^. Further evidence of single molecule pulling is demonstrated by estimating the contour length increase resulting from unfolding events, which was found to be uniform (Supplementary Fig. S6). Thus, multi-protein pulls, if they exist, contribute only minimally to the data we report.

**Figure 3:**
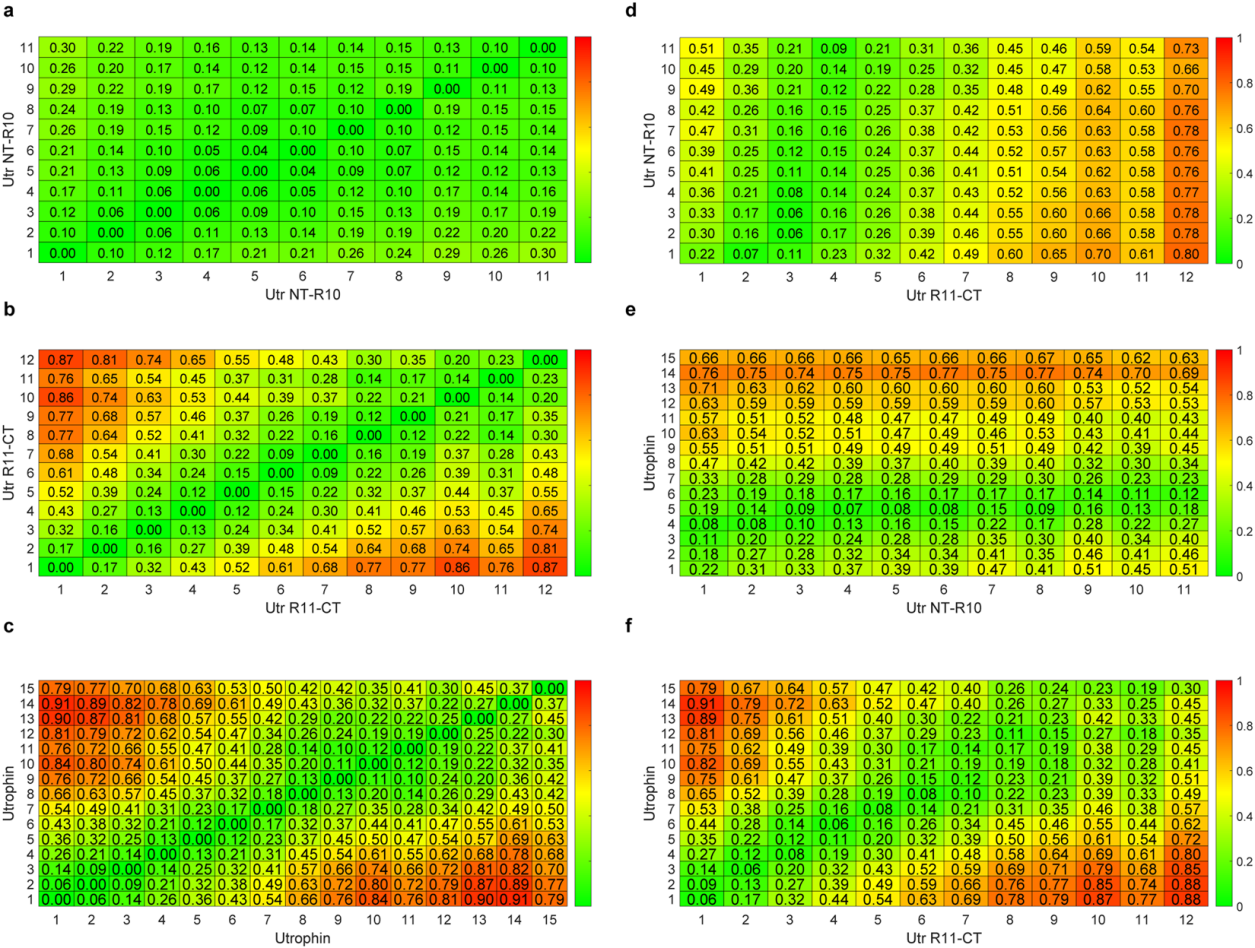
Kolmogorov-Smirnov (KS) test metrics. KS test metrics which compare the distribution of unfolding forces for different unfolding event counts. The color-bars (heat maps) represent the KS metric with a 0 value (green) indicating similarity and a value of 1 representing maximum dissimilarity (red). Each of the horizontal and vertical axes represents unfolding event counts from the corresponding molecule. The sub-figures (**a**–**c**) compare the distributions within the same molecule (self-comparison). The sub-figures (**d**–**f**) with horizontal and vertical axes from different molecules demonstrate cross-comparisons.

To determine statistical consistency, we collected data for 300–500 successful pulling experiments (with at least one identifiable unfolding event) for each protein construct and analyzed distributions for unfolding force, contour length increment, and persistence length (Supplementary Figs S4 and S7–8. The overall unfolding force distributions of the utrophin constructs do not show significant differences (Supplementary Fig. S4a–c). However, distributions of the unfolding force as a function of the unfolding event count within individual force traces revealed different behaviors for Utr NT-R10 and Utr R11-CT (Fig. 2). For Utr NT-R10, the statistics of the unfolding forces for different unfolding event counts were similar with overlapping distributions (Fig. 2a), where the unfolding force distribution for the first unfolding event is similar to all unfolding events between the second and the eighth, demonstrating a brittle behavior. Force distributions for Utr R11-CT (Fig. 2b) and full-length utrophin (Fig. 2c) were right-shifted and broadened as the unfolding event increased, demonstrating a stiffening spring behavior. To quantify these differences, we performed the Kolmogorov-Smirnov (KS) test that gives a measure of similarity between two distributions (Fig. 3)^19^. A KS distance of zero (green) between a pair of distributions indicates identical distributions and a value of one (red) indicates maximally different distributions. The results of this statistical test comparing the distribution of each unfolding event with every other unfolding event within each protein construct and across protein constructs demonstrate that the unfolding behavior of Utr NT-R10 is statistically different from Utr R11-CT and full-length utrophin. Here, the maximum KS distance between the 1st unfolding event and any other unfolding event within Utr NT-R10 (Fig. 3a) is 0.30 in contrast to a maximum KS distance of 0.87 and 0.91 for Utr R11-CT and full-length utrophin, respectively (Fig. 3b,c). The KS tests also show that all the unfolding events of Utr NT-R10 have similar distributions of forces for unfolding (Fig. 3a) whereas Utr R11-CT and utrophin force distributions become more dissimilar with increasing unfolding events (Fig. 3b,c). KS test cross comparisons between different utrophin constructs demonstrate that the distributions of Utr NT-R10 exhibit increasing dissimilarity with increasing unfolding events compared to Utr R11-CT and full-length utrophin as indicated by heat maps (Fig. 3d,e). In contrast, the heat map comparing Utr R11-CT and full-length utrophin demonstrates similar distributions with increasing unfolding events (Fig. 3f). Overall, these data show uniform unfolding forces upon extension for Utr NT-R10 and a nonlinear increase in unfolding forces upon extension for Utr R11-CT and full-length utrophin. The mechanical differences demonstrated between Utr NT-R10 and Utr R11-CT are in striking contrast to previously published circular dichroism data showing nearly identical thermal melt profiles for Utr NT-R10 and Utr R11-CT^20^.

**Figure 4:**
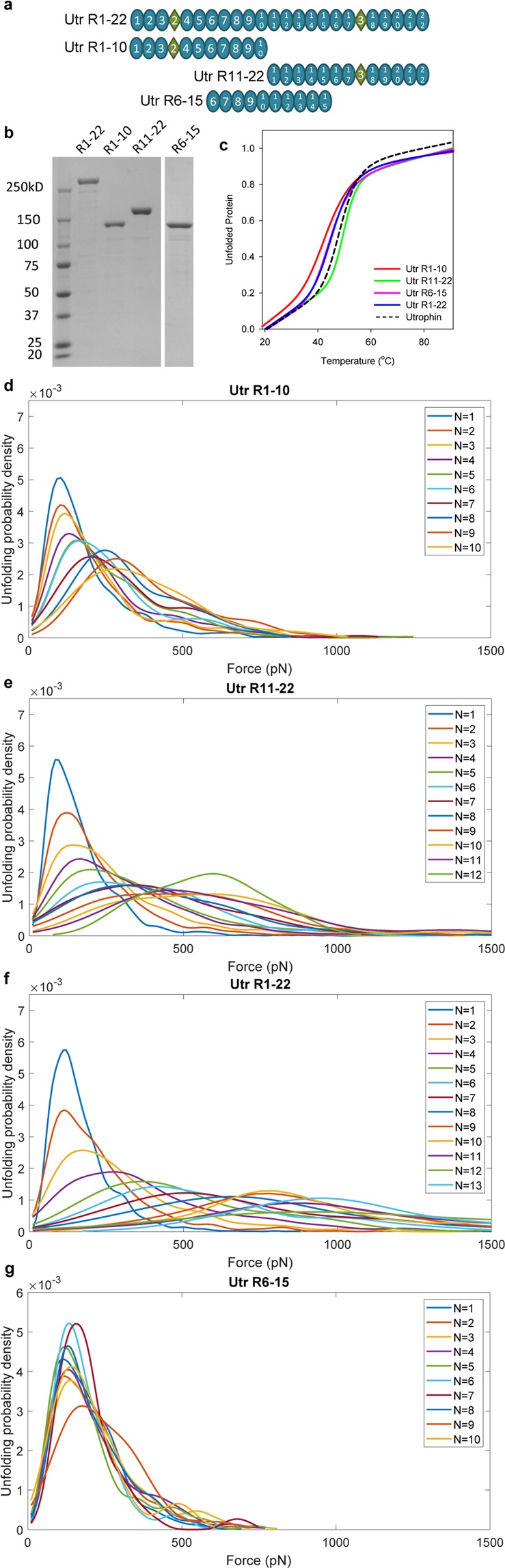
Unique mechanical behavior of utrophin halves is maintained upon deletion of terminal domains. (**a**) Schematic of utrophin constructs lacking terminal domains and a 10-repeat construct spanning both N- and C-terminal halves. (**b**) Coomassie-stained gel of purified proteins (5ug) imaged on UVP GelDoc-It® Imaging System. (**c**) Circular dichroism melt curves of utrophin constructs measured at 1 °C intervals. Average melting temperatures ± standard deviation for Utr R1-10, R11-22,R1-22, and R6-15 are 45.7 ± 0.26 °C, 50.5 ± 0.3 °C, 47.0 ± 0.49 °C, and 45.4 ± 1.6, respectively. (**d**–**f**) Plots of probability distributions vs unfolding force of each unfolding event (N) for Utr R1-10 (**d**), Utr R11-22 (**e**), Utr R1-22 (**f**), and Utr R6-15 (**g**).

One possible explanation for the different mechanical properties of Utr NT-R10 and Utr R11-CT is that their 10–12 homologous spectrin-like repeats are controlled by long-range intra-protein communication from the unique ABD1 and/or CRCT terminating domains. To test the possibility of long-range effects we expressed and purified three new utrophin constructs (Fig. 4a,b) by deleting ABD1 (Utr R1-10), CRCT (Utr R11-22) or both ABD1 and CRCT (Utr R1-22). Additionally, we expressed a construct containing 10 spectrin-like repeats that span both the N-terminal and C-terminal halves (Utr R6-15). Circular dichroism spectroscopy revealed similar thermal melt profiles for all four constructs (Fig. 4c). In the absence of terminal ABD1 and/or CRCT domains, Utr R-10, Utr R11-22, and Utr R1-22 constructs maintained the brittle, or stiffening behaviors (Fig. 4d–g) observed with the original terminal domain-containing constructs (Figs 2–3). Application of the Kolmogorov-Smirnov test to compare the unfolding force distributions with Utr NT-R10, Utr R11-CT, and full-length utrophin confirm that the termini do not significantly contribute to the mechanical behavior of utrophin (Supplementary Figs S9–10). Interestingly, Utr R6-15 displays brittle mechanical behavior despite containing 5 spectrin-like repeats associated with the stiffening-spring behaving C-terminal half of utrophin.

**Figure 5:**
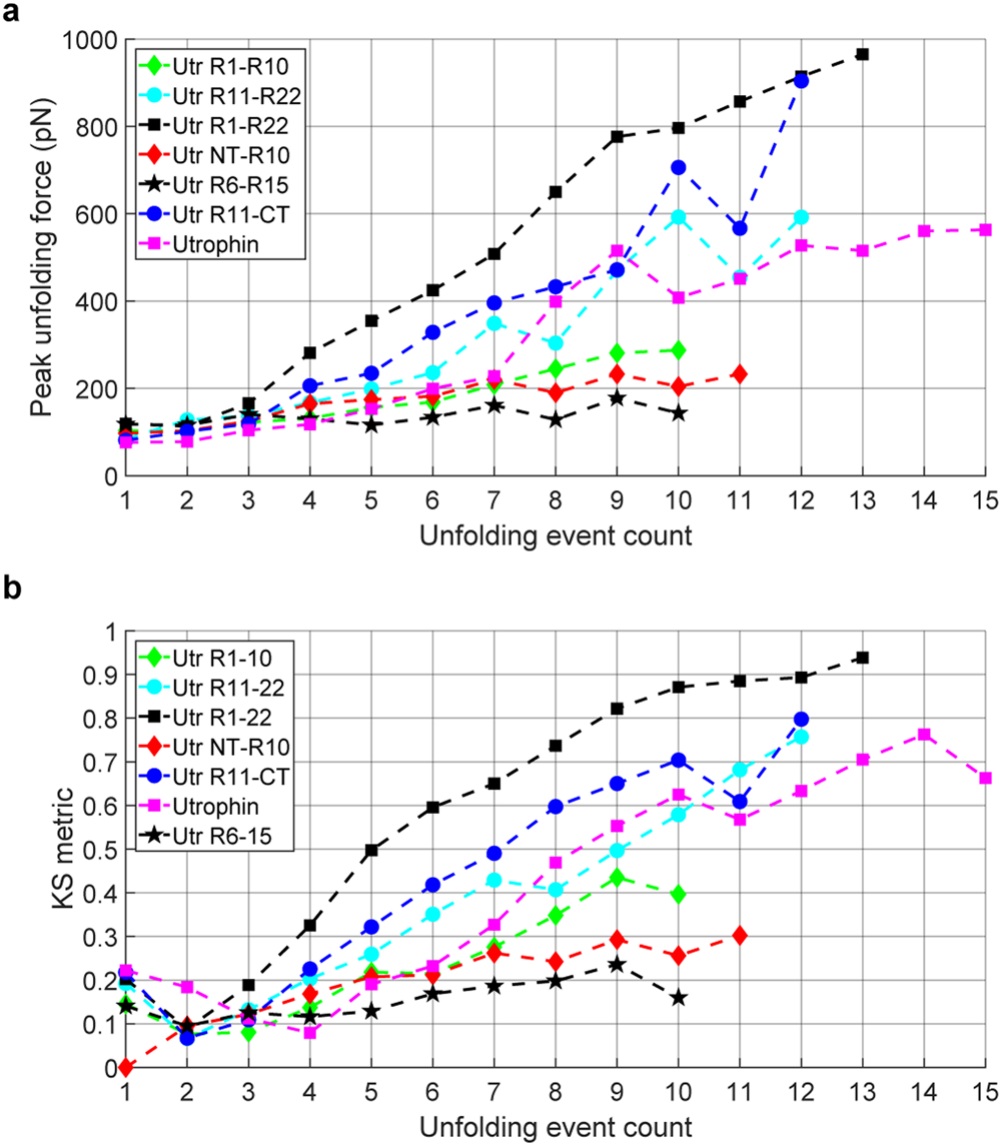
Summary of utrophin mechanical behavior. (**a**) Comparison of modes of the unfolding force distributions vs unfolding event count shows brittle vs stiffening behavior of utrophin constructs. Peak (or mode) of the distribution of unfolding forces expressed as a function of the number of domains unfolded (unfolding event count). Data are combined over 3 batches of 300–500 successful pulling experiments per batch for each protein construct and more detailed statistics are provided in Supplemental Fig. S11. (**b**) KS metrics for the distributions of unfolding force corresponding to different unfolding event counts from each of the utrophin constructs. The KS metrics are computed by using the force distribution of the first unfolding event of Utr NT-R10 as the reference distribution.

To summarize the AFM results, we plotted the peak (or mode) of the distribution of unfolding forces as a function of unfolding events obtained from 300–500 successful pulling experiments for each of the six utrophin constructs (Fig. 5a, Supplementary Fig. S11). Figure 5b provides a plot of the KS distance for every unfolding event compared to the distribution of the first unfolding event distribution of Utr NT-R10 and demonstrates the statistical difference between the N-terminal half (red and green plots) and C-terminal half (blue and cyan plots) of utrophin independent of their unique terminating domains (ABD1 and CRCT, respectively). Despite their high sequence and structural homology, our results suggest the sequences of spectrin-like repeats within the N-terminal and C-terminal halves of utrophin encode information that dramatically influences their respective mechanical behaviors. Additionally, Fig. 5 reveals that full-length utrophin, both with and without its terminating domains (pink and black plots), exhibit mechanical behavior directed by the C-terminal half whereas a construct containing both N-terminal and C-terminal half spectrin-like repeats exhibits mechanical behavior directed by the N-terminal half (Utr R6-15, black star plot).

**Figure 6:**
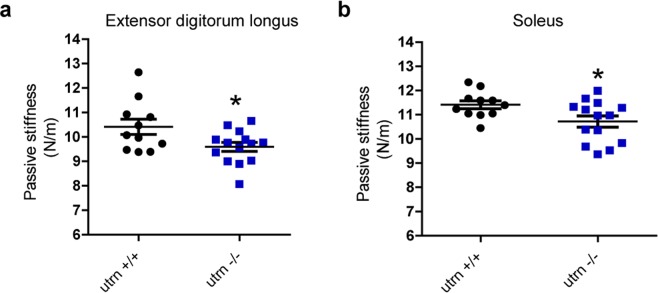
*Ex vivo* analysis of passive stiffness in utrophin null mice. Passive stiffness measurement of extensor digitorum longus (EDL) (**a**) and soleus (**b**) muscle of wild-type (utrn+/+) and utrophin null (utrn−/−) mice. Data presented as individual points with mean and standard error of the mean (S.E.M); unpaired t-test, *p < 0.05, compared to utrn+/+. For utrn+/+, n = 11 and for utrn−/−, n = 14. Cohen’s d between utrn+/+ and utrn−/− is 0.95 for EDL, and 0.94 for soleus.

The stiffening spring behavior and high forces of unfolding in utrophin are similar to the mechanical properties of titin rather than spectrin (Supplementary Fig. S1–2). The biological implication of these findings is that utrophin may be too stiff to be functioning as a spring at the sarcolemma. Rather, the mechanical properties of utrophin may be suited to function as part of an in-series elastic element with titin at the myotendinous junction where it is localized in adult skeletal muscle^9,21^. To test this hypothesis, we measured the passive stiffness of extensor digitorum longus (EDL) and soleus muscles from utrophin null mice^22,23^. We chose the EDL muscle because it is most frequently characterized in *ex vivo* physiological studies on muscular dystrophy while the soleus was chosen because utrophin expression is >3-fold higher in mouse soleus than in EDL^24^. Consistent with our hypothesis, both EDL and soleus muscles from utrophin null mice exhibited significantly lower passive stiffness compared to wild-type (Fig. 6). No other contractility measures differed between muscles with and without utrophin (Supplementary Table S1). These passive stiffness data together with our AFM measurements suggest that utrophin may be functioning in series with titin as part of a passive elastic element at the myotendinous junction in non-contracting muscle.

## Discussion

In comparison to single molecule force spectroscopy data for other proteins containing spectrin-like repeats^10–12^, our results for utrophin reveal much higher forces of unfolding and stiffening behavior which is similar to the mechanically stiff spring titin^25,26^. The consistently linear increase in the contour length with increasing unfolding events (Supplementary Fig. S6) and contour length increment values that are consistent with single repeats (Supplementary Fig. S7) argues against the possibility that the high unfolding forces observed for utrophin are due to simultaneous unfolding of multiple repeats. Statistics of the elongation of a molecule between successive unfolding events provide important insights and have revealed the presence of intermediate domains in previous studies^27–29^. Such intermediate pathways will result in multiple peaks and can shift the distribution of unfolding forces. However, for the proteins we investigated, the statistics of elongation between successive unfolding events exhibit a single peak (or mode) of distribution (Supplementary Fig. S12). Moreover, intermediate unfolding events are likely to result in changes in the contour length increment associated with each event, but such changes are not observed in the data (Supplementary Figs S6–7). Thus, we find no evidence of intermediate unfolding pathways as described previously for other molecules^26–28^.

It is more likely that the differences in the measured forces of unfolding of spectrin repeat-containing proteins are influenced by sequence differences. Of the spectrin family of proteins, dystrophin and utrophin exhibit lower sequence similarity in comparison to the other family members (alpha-actinin, alpha-spectrin, and beta-spectrin)^30^. Moreover, dystrophin and utrophin have much weaker conservation between repeat units, with a lower number of conserved residues and greater number of insertions compared to repeats within the spectrins^31^. In addition to differences in mechanical behavior between spectrin repeat-containing proteins, our data are the first to demonstrate markedly different mechanical behaviors for structurally homologous spectrin-like repeats within the same molecule, a finding that warrants future direct comparisons between full-length dystrophin and utrophin and analogous fragments.

*In vivo* mechanical studies of plasma membrane adhesion complexes such as integrins and cadherins have revealed unfolding forces that are within the ~25 pN range of those measured for repeats in spectrin *in vitro*^15,32,33^. Three previous mechanical studies on dystrophin central rod fragments using AFM and optical tweezers reveal similar and even smaller forces of unfolding (7–40 pN range), values that are in line with dystrophin acting as a molecular spring at the sarcolemma^10–12^. The surprisingly high unfolding forces of utrophin measured here, particularly in Utr R11-CT and full-length utrophin, suggest that utrophin is too stiff to function as a spring that protects the sarcolemma from mechanical stress. Alternatively, the stiffness of utrophin is more consistent with its localization to the myotendinous junction (MTJ)^9^, the primary site of muscle force transmission to bone, where titin acts as an elastic element in the development of passive force^34^. By AFM, Ig domains of titin display increasing unfolding forces upon extension^26^ similar to what we observed for Utr R11-CT and full-length utrophin. AFM measurements for the PEVK domain of titin show unfolding forces that are uniform upon extension^35^, a property that is comparable to what we observed for the actin binding half of utrophin (Utr NT-R10). Interestingly, the PEVK domain of titin also binds actin. Based on our observations, we hypothesized that utrophin may function in series with titin as a restorative elastic element. To address our hypothesis, we generated utrophin null mice and measured passive stiffness in EDL and soleus muscles, a parameter associated with titin elasticity^36^. Utrophin null mice have been previously assessed for several parameters but initial physiological assessments did not reveal a significant muscle phenotype^22,23^. A mouse line deficient for both utrophin and α7 integrin was previously demonstrated to have defects in the myotendinous junction but any conclusions about the unique role of utrophin were confounded by an α7 integrin phenotype^21^. We measured a significant decrease in soleus and EDL passive stiffness in utrophin null mice, demonstrating a novel phenotype associated with utrophin deficiency. Together, our mechanical assessment of utrophin by AFM and our physiological analysis of mice lacking utrophin support our hypothesis that utrophin functions as a stiff elastic element in series with titin at the myotendinous junction in adult skeletal muscle.

## Methods

### Cloning

Full-length mouse utrophin was previously cloned from an existing vector into a pENTR/D-TOPO vector (Invitrogen™) with an 8-amino acid FLAG-tag (DYKDDDDK) added to the N-terminus of utrophin for use in purification^20^. All utrophin truncation constructs were PCR amplified using primers designed around adjacent repeats for the desired deletion based on reported repeat and domain boundaries^31^. The PCR products were circularized using T4 polynucleotide kinase and T4 DNA ligase (New England BioLabs) and sequence verified. Using the Gateway Recombination system (Life Technologies), the deletion constructs were recombined into the pDEST8 destination vector and subsequently transformed into DH10Bac competent *E*. *coli* and purified according to manufacturer’s protocol.

### Protein expression and purification

Sf9 insect cells were maintained at 1e10^6^ cells/mL in Sf-900™ II SFM (Life Technologies). Purified Baculovirus was transfected using Cellfectin^®^ II (Life Technologies) and high-titer viral stocks were generated through successive infections of Sf9 cells in 3.5 cm plates (P0), 10 cm plates (P1), and 250 mL of 1e10^6^ cells/mL suspended cells (P2). Ten mL of P2 virus were used to infect 250 mL of 1e10^6^ cells/mL and cultured for 72-hour post-infection to maximize protein expression. Infected cells were centrifuged at 1,000 × *g* for 3 minutes and re-suspended in lysis buffer containing phosphate buffered saline (PBS), 1% Triton, and a cocktail of protease inhibitors [100 nM Aprotinin, 10 μM E-64, 10 μM Leupeptin, 1 mM PMSF, 1 μg/mL Pepstatin, 1 mM Benzamidine]. The lysate was centrifuged at 14,000 × *g* for 10 min at 4 °C and the supernatant applied to an anti-FLAG M2 agarose column (Sigma Aldrich). The column was washed with >10 column volumes of PBS and bound protein was eluted with PBS containing protease inhibitors and 100 μg/mL FLAG peptide. After dialysis overnight in 2 L of PBS at pH 7.5, the purified protein was concentrated using the Amicon Centrifugal Filter unit (UFC801024) and protein concentration was determined using A_280_ and extinction coefficients calculated from the amino acid compositions for each construct. For each utrophin construct, purification fractions and concentrate were run on a 3–12% SDS polyacrylamide gradient gel at 150 V for 1 hour. The gels were stained with Coomassie blue stain, de-stained, and visualized using the UVP GelDoc-It® Imaging System. For Fig. 4b, utrophin constructs were purified in parallel (with the exception of Utr R6-15) and 5 µg of concentrated protein were visualized on the same gel as described. Upon initial purification, Utr NT-R10 showed a higher molecular weight band indicating oligomerization. Thus, 0.5 mM dithioreitol was added to the elution and dialysis buffers resulting in a band at the appropriate molecular weight (166 kDa, data not shown). None of the other constructs demonstrated oligomerization.

### Circular Dichroism

Each purified protein was centrifuged at 14,000 *g* for 10 minutes at 4 °C and the supernatant diluted to 0.4 mg/mL using PBS. Absorption spectra were acquired with a Jasco J-815 spectropolarimeter, initially at 20 °C as controlled by a Peltier device, from 200 to 260 nm wavelength with a scanning rate of 50 nm/min. Spectra were then acquired at a temperature gradient of 1 °C/min temperature intervals from 20–90 °C and the characteristic ellipticity at alpha-helical wavelength (θ_222_) recorded. Molar ellipticity, [θ], was calculated using the following equation: [θ] = θ/(10*cl)* where c is the molar concentration of the sample (mole/L) and *l* is the path-length in cm. Molar ellipticity (with units of degrees, cm squared per decimole) was plotted against wavelength for the circular dichroism (CD) spectra. Ellipticity at 222 nm (θ_222_) was normalized, plotted against temperature, and fit by regression analysis in Sigma Plot (Systat Software, Inc.) using equations for two state unfolding^20^. For Utr R1-22, R1-10, R11-22, and R6-15 constructs, each protein was analyzed by circular dichroism after three separate protein preparations. The average standard errors of estimate for Utr R1-22, R1-10, R11-22, and R6-15 fits were 0.014, 0.013, 0.015, and 0.010 respectively. The mean melting temperatures calculated from the two state unfolding equation and standard deviations are listed in Fig. 4.

### Atomic Force Microscopy

The single molecule force spectroscopy experiments were performed utilizing a MFP-3D atomic force microscope (AFM) from Oxford Instruments. The AFM setup contained a flexible cantilever with a sharp tip, a laser-photodiode based sensor which measures the position of the cantilever tip, and a piezo electric nano-positioner which can move the substrate in three spatial directions with respect to the cantilever base^37^. We used a soft BioLever (BL-RC-150VB) from Asylum Research with a typical spring constant of 6 pN/nm and a tip radius of 25 ± 12 nm coated with Cr/Au. A droplet (~100 µl) of purified protein solution (at concentrations ranging from 12.5–200 nM) was deposited on freshly cleaved mica substrate and allowed to settle for 10 minutes before commencing the experiment to ensure adsorption of proteins to the mica surface. The cantilever tip was made to approach the mica surface, pressed against the surface for 3 seconds with a force ranging from 1000–2000 pN and then retracted with a specified velocity. For the experiments on the utrophin constructs, a retraction velocity of 1 *μms*^−1^ was used. The approach-retraction cycle was repeated multiple times. During such a cycle, if a part of a protein molecule was attached to the cantilever tip with another part adsorbed on the substrate, the intermediate segment was stretched during the retraction phase of the cantilever. The pulling force on the molecule was balanced by the force on the cantilever which is estimated from the product of the deflection of the cantilever tip and the spring constant of the cantilever. The spring constant of the cantilever was determined before the pulling experiments by analyzing the thermal response of cantilever deflection^38^. Forced extension caused the folded domains in the molecule to unfold, characterized by the saw-tooth pattern observed in the cantilever deflection vs separation curve^15^. To eliminate possibilities of pulling on multiple proteins, initial force extension data were monitored for instances where the number of unfolding events exceeded available domains for a single molecule. The sample was diluted until the number of unfolding events remained in the expected range (See Supplementary Fig. S13). Additionally, the success rate of pulling was maintained between 5–25%. Data from 300–500 successful force spectroscopy experiments (with at least one identifiable unfolding event) were collected for each protein construct which were used to determine the statistical behavior of the unfolding forces.

### Data analysis

Data from each of the successful protein pulling experiments were collected and analyzed using the MATLAB software from MathWorks^®^. Collected data included the separation of the cantilever base from the surface of the substrate from which the extension of the molecule was calculated. Data also included the measured deflection of the cantilever tip as a function of separation. Since the cantilever deflection is proportional to the force applied, the measured deflection can be converted to the force acting on the cantilever by multiplying the spring constant of the cantilever. Any observed force peak in the data was required to be greater than three standard deviations from the approach curve (3σ) to be considered a true unfolding event. The first significant peak is corrupted by the adhesion forces between the tip of the AFM cantilever and the substrate/protein and thus was discarded. Similarly, the last significant peak was not considered for analysis as it most likely represents the detachment of the protein molecule from the tip of the cantilever. The extension of protein molecules in between successive unfolding events was fit with the worm like chain (WLC) models. The WLC model^15^, which relates the force (*F*) exerted on the molecule to its extension (*x*) is shown in Eq. 1.

1 $$F(x)=\frac{{k}_{B}T}{P}{\textstyle (}\frac{1}{4{(1-\frac{x}{L})}^{2}}-\frac{1}{4}+\frac{x}{L}{\textstyle )}.$$

Parameters of the model are the persistence length (*P*) and the contour length (*L*), with statistics obtained for each utrophin construct (see Supplementary Figs S7–S8), *T* is the temperature and *k*_*B*_ is the Boltzmann constant. As a validation of our experimental setup and data analysis protocols, we obtained the statistics of the persistence length and contour length for the protein titin I27O. Values of the most likely unfolding force, contour length, and persistence length were found to be 212 pN, 26.4 nm, and 304 pm respectively, which matched well with the reported values^14^ of 225 pN, 28.4 nm, and 390 pm, respectively (see Supplementary Fig. S1).

To further lessen the impact of measurement noise and to remove chances of analysis and conclusions getting corrupted by pulling of multiple protein strands the analysis was repeated on filtered data. Three filters of increasing strengths, Type A, B, and C, based on the expected contour length were used. A single force-extension curve considered for analysis consisted of one or more unfolding events. The contour length increment for unfolding event *i* is defined as ${\rm{\Delta }}{L}_{{c}_{i}}:\,={L}_{{c}_{i}}-{L}_{{c}_{i-1}},$ where ${L}_{{c}_{i}}$ is the contour length before unfolding event *i* occurs, measured in nm. Events with a negative contour length increment were removed from further analysis even when filters were not used. The Type A filter discards an entire force-extension curve if it contained at least one event with a negative contour length increment estimate. The Type B filter, in addition Type A filtering, removes any events whose estimated contour length was outside expected bounds. The Type C filter, in addition to Type A filtering, also discards the entire force-extension curve if it contained at least one event with estimated contour length falling outside expected bounds. The expected contour length contribution for a domain was taken as the product of the number of amino acid residues in the domain, which is known, with the average contour length contribution of a single residue (0.299 nm^15^). For the first unfolding event, the smallest domain (with the smallest number of amino acids) in the molecule formed the lower bound of the contour length estimate, and the largest domain, the upper bound. Similarly, for the second unfolding event, the lower bound was obtained from the two smallest domains in the molecule, and the upper bound from the two largest domains. The contour length bounds for the subsequent n^th^ event were similarly computed from the contour length contributions of the n smallest and n largest domains in the molecule. We further applied a tolerance factor of 50% to the bounds obtained from expected contour length increments to accommodate the errors in the parameters’ estimates due to measurement noise. The presence of unstructured regions within the central rod domain of utrophin, termed “hinges”, are not well characterized and it is unknown whether these domains contribute to unfolding events. In our filtering process, we used the expected contour length increments of these hinge regions ranging from 5.4–22.8 nm as the lower limit, which includes the traditional hinge regions 1–4 depicted in the schematic (Fig. 1a) as well as an unstructured region between repeats 13 and 14^31^. The filtered data lead to the same results as the unfiltered data, further reinforcing our conclusions (see Supplementary Figs S14–S16).

### Kolmogorov-Smirnov test analysis

The sample distributions $({D}_{i}=\{{f}_{1},{f}_{2},\ldots ,{f}_{{n}_{i}}\})$ of unfolding forces of different unfolding events were compared using the Two Sample Kolmogorov-Smirnov (KS) test. When comparing two sample distributions *D*_*i*_ and *D*_*j*_, the KS metric (*H*_*KS*_) is a measure of the distance between them^19^. *H*_*KS*_ is given by Eq. 2,

2 $${H}_{KS}^{\,}=\mathop{max}\limits_{f}|{S}_{i}(f)-{S}_{j}(f)|\,,$$

where, *S*_*i*_(ƒ) is the empirical distribution function of the sample force distributions (*D*_*i*_) defined by: ${S}_{i}(f)=\frac{1}{{n}_{i}}\sum _{k=1}^{{n}_{i}}{{\mathbb{I}}}_{{f}_{k}\le f},$ where, *n*_*i*_ is the total number of elements in *D*_*i*_. Here ${{\mathbb{I}}}_{{f}_{k}\le f}$ is the indicator function defined by:

$${{\mathbb{I}}}_{{f}_{k}\le f}\,:\,={\textstyle \{}\begin{array}{l}1\,{\rm{i}}{\rm{f}}\,{f}_{k}\le f\\ 0\,{\rm{o}}{\rm{t}}{\rm{h}}{\rm{e}}{\rm{r}}{\rm{w}}{\rm{i}}{\rm{s}}{\rm{e}}\end{array}\,,$$

*H*_*KS*_ ranges from 0 to 1, with 0 indicating identical distributions, and 1 indicating maximally dissimilar distributions.

### Generation of mice

The following breeding scheme was used to generate utrophin-null mice. First, males genotyped X^mdx^Y; Utr^+/−^ were crossed with C57/Bl10 females. Resulting male F1 offspring with the genotype X^wt^Y; Utr^+/−^ were crossed with female F1 offspring with the genotype X^wt^X^mdx^; Utr^+/−^. To maintain a utrophin-null line, male F2 offspring with the genotype X^wt^Y; Utr^−/−^ were crossed with female F2 offspring with the genotype X^wt^X^wt^; Utr^−/−^. All animals were housed and treated in accordance with the standards set by the University of Minnesota Institutional Animal Care and Use Committee.

### Mouse physiology

Mice at three months of age were anesthetized with 100 mg/kg body mass sodium pentobarbital and extensor digitorum longus (EDL) and soleus muscles dissected. Silk suture was used to attach the distal tendon to a static structure and the proximal tendon to a force transducer (Model 300B-LR, Aurora Scientific). The EDL or soleus was incubated in Krebs-Ringer bicarbonate buffer [120.5 mM NaCl, 4.8 mM KCl, 1.2 mM MgSO_4_ 1.2 mM Na_2_HPO_4_, 20.4 mM NaHCO_3_, 10 mM glucose, 10 mM pyruvate, 1.5 mM CaCl_2_], oxygenated with 95% O_2_/5% CO_2_. Muscles were set to their anatomic length (L_o_) which was measured from myotendonous junction to myotendonous junction using digital calipers. Muscles remained quiescent for 5 min before passive stiffness was determined by stretching non-contracting muscles sinusoidally from 97.5% L_o_ to 102.5% L_o_ at 0.5 Hz while measuring the resulting force^39,40^. Isometric tetanic contractions separated by 2 min followed 30 s later until a plateau was attained (within 5 mN). EDL muscles were stimulated for 200 ms at 175 Hz and soleus muscle for 400 ms at 120 Hz (Grass S48 stimulator delivered through a SIU5D stimulus isolation unit; Grass Telefactor, Warwick, RI). Active stiffness was measured from a sinusoidal length oscillation of 0.01% at 500 Hz during a tetanic isometric contraction^39,40^. Statistical analysis for physiological measurements included an unpaired t-test (GraphPad Prism 5) and Cohen’s d metric calculation for effect size (Cohen’s d for samples $\{{x}_{1},\ldots ,{x}_{n}\}$ and $\{{y}_{1},\ldots ,{y}_{m}\}$ are given by $d=\frac{\bar{X}-\bar{Y}}{\sigma }$; where $\bar{X}=\frac{{\sum }_{i=1}^{n}{x}_{i}}{n}$ and $\bar{Y}=\frac{{\sum }_{i=1}^{m}{y}_{i}}{m}$ are the sample means, and $\sigma =\sqrt{\frac{{\sum }_{i=1}^{n}{({x}_{i}-\bar{X})}^{2}+{\sum }_{i=1}^{m}{({y}_{i}-\bar{Y})}^{2}}{n+m-2}}$, is the pooled standard deviation)^41^. All animal experiments were approved by the University of Minnesota Institutional Animal Care and Use Committee under protocol numbers 1506–32699A, and 1806–36018A.

## Supplementary information

Supplementary Figures

## Data Availability

The datasets analyzed during the current study are available from the corresponding author on reasonable requests.
