# Supplementary material for: Distinct mechanical properties in homologous spectrin-like repeats of utrophin
Source: Sci Rep. 2019 Mar 26;9:5210. doi: 10.1038/s41598-019-41569-4 (PMC6435810; doi:10.1038/s41598-019-41569-4)
Supplement: Supplementary file 1 — Supplementary Figures [file 41598_2019_41569_MOESM1_ESM.pdf]

Distinct mechanical properties in homologous spectrin-like repeats of utrophin

Sivaraman Rajaganapathy[1#], Jackie L. McCourt[2#], Sayan Ghosal[1#], Angus Lindsay[3], Preston M. McCourt[2], Dawn A. Lowe[3], James M. Ervasti[2*], and Murti V. Salapaka[1]

#These authors contributed equally to this work

*Corresponding author

[1]Department of Electrical and Computer Engineering, University of Minnesota – Twin Cities, Minneapolis, MN 55455.

[2]Department of Biochemistry, Molecular Biology, and Biophysics, University of Minnesota – Twin Cities, Minneapolis, MN 55455.

[3]Department of Rehabilitation Medicine, University of Minnesota – Twin Cities, Minneapolis, MN 55455.

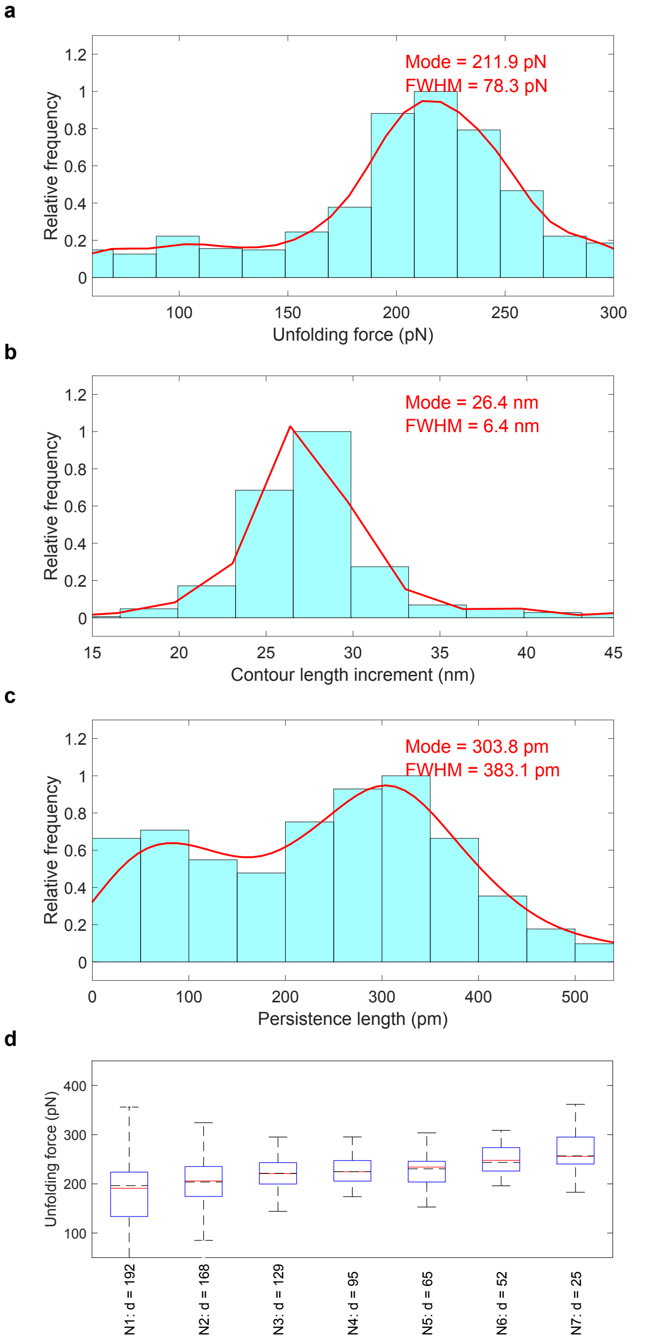

Supplementary Figure S1. AFM statistics of titin I27O (an AFM reference protein with 8 repeats of the Ig 27 domain of human titin). **a**, Histogram plot of unfolding forces with the mode at 212 pN when pulled at 1um/s with a cantilever spring constant of 6.67 pN/nm. This matches closely with the reported value of 225 pN[14]. **b**, Histogram plot of contour length increments indicates that the most probable value is 26.4 nm, while 28.4±0.3 nm was the corresponding reported value[14]. **c**, The peak persistence length was measured to be 304 pm compared to the reported value of 390±70 pm[14]. **d**, Box plots of the unfolding forces classified based on the unfolding count, with the red line indicating the median value and the dotted black line indicating the mode. The edges of the box represent the 25% and 75% percentiles, with the whisker plots marking the minimum and maximum recorded values excluding the outliers. The unfolding forces for titin I27O range from 200 pN to 300 pN (a 50% increase) for unfolding counts of 1 to 8, respectively.

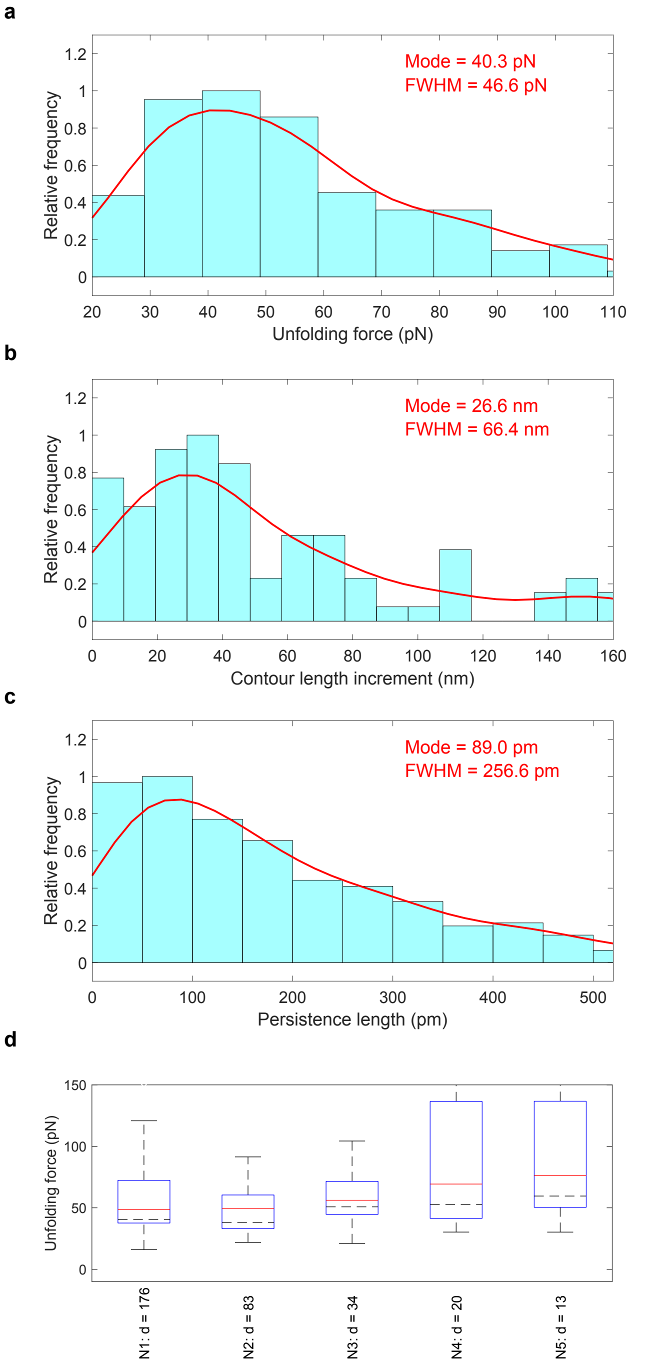

Supplementary Figure S2. AFM statistics of spectrin. a, Unfolding force histogram when pulled at 0.25 um/s with a cantilever of spring constant 6.88 pN/nm with a most likely peak value of 40 pN compared to 28 pN reported in the literature under similar conditions[15]. **b**, Contour length increment histogram plot with a peak value of 26.6 nm, consistent with a value of 31.7 nm from the earlier report[15]. **c**, Persistence length histogram plot with a peak measured 89 pm. **d**, Box plots of the unfolding forces classified based on the unfolding count, with the red line indicating the median value and the dotted black line indicating the mode. The edges of the box represent the 25% and 75% percentiles, with the whisker plots marking the minimum and maximum recorded values excluding the outliers. The unfolding forces vary from 26 pN for the first unfolding event to 40 pN for the sixth (a 54% increase), indicating that the molecule exhibits a mildly stiffening spring behavior.

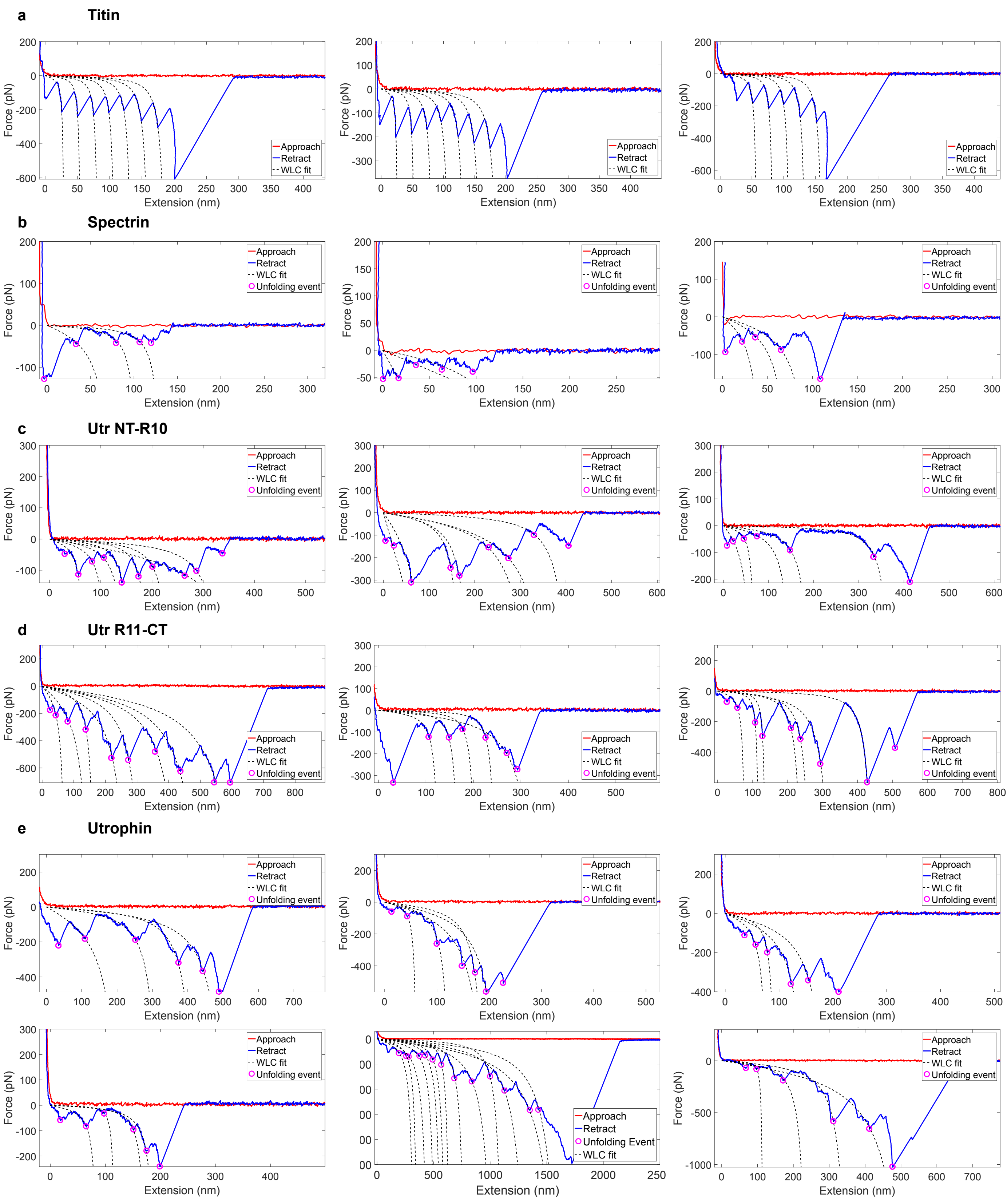

Supplementary Figure S3. Additional force vs extension curves with WLC fits. Candidate force vs extension curves of titin (**a**), spectrin (**b**), Utr NT-R10 (**c**), Utr R11-CT (**d**), and full-length utrophin (**e**). Approach - force on the cantilever as it approaches the substrate; retract - force on the cantilever as the molecule is extended; WLC fit - fit of the worm like chain model, which relates the force exerted on the molecule to its extension; unfolding event - force minima corresponding to domain unfolding.

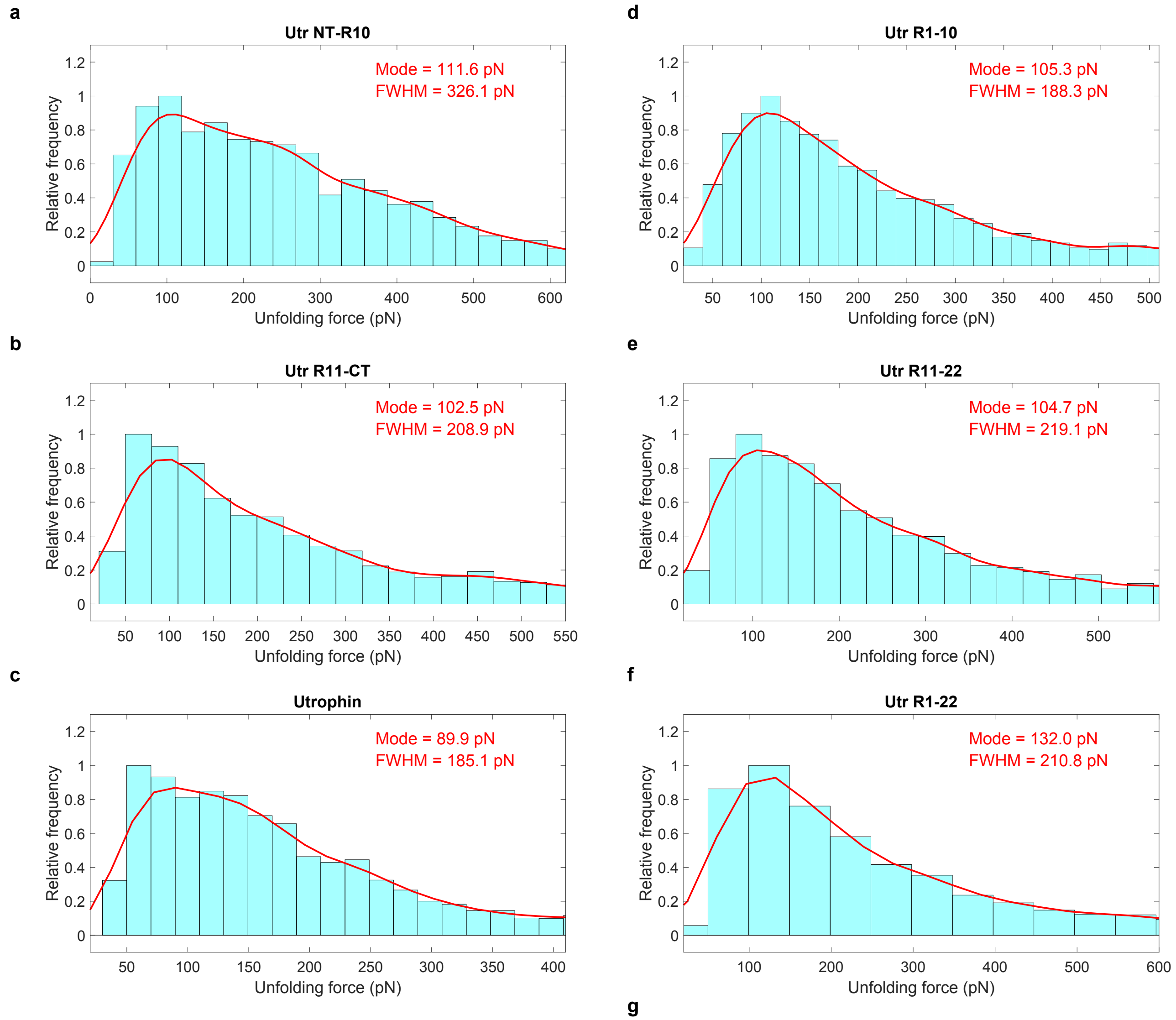

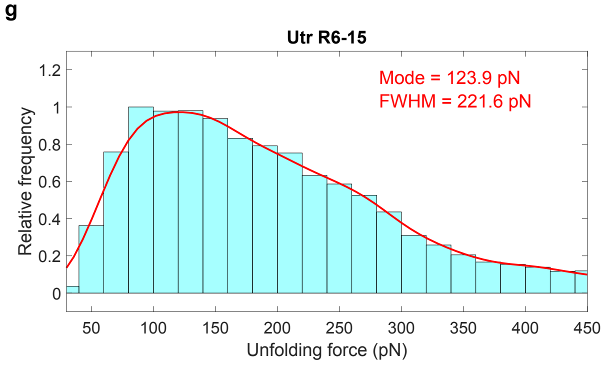

Supplementary Figure S4. Unfolding force histograms for the seven utrophin constructs. The unfolding forces are reported for a pulling speed of 1 um/s with cantilever spring constants between 5.04-9.06 pN/nm.

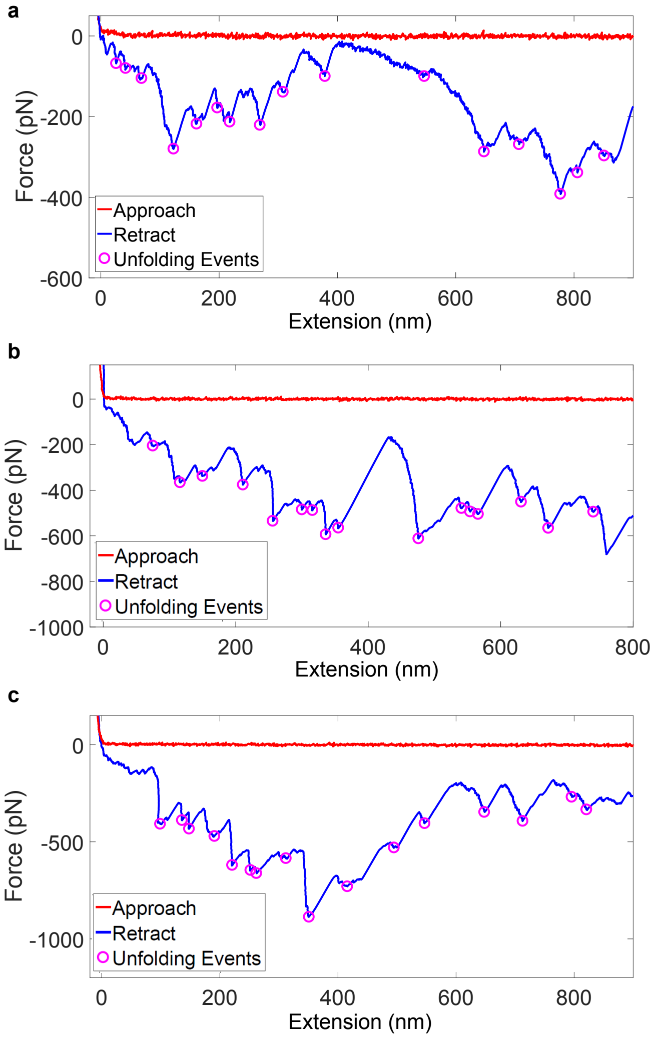

Supplementary Figure S5. Force extension characteristics of multiple protein pulls. a-c, Example force traces of Utr NT-R10 at high concentrations (500nM) with unfolding events exceeding the number of available domains for a single molecule (11 domains for Utr NT-R10).

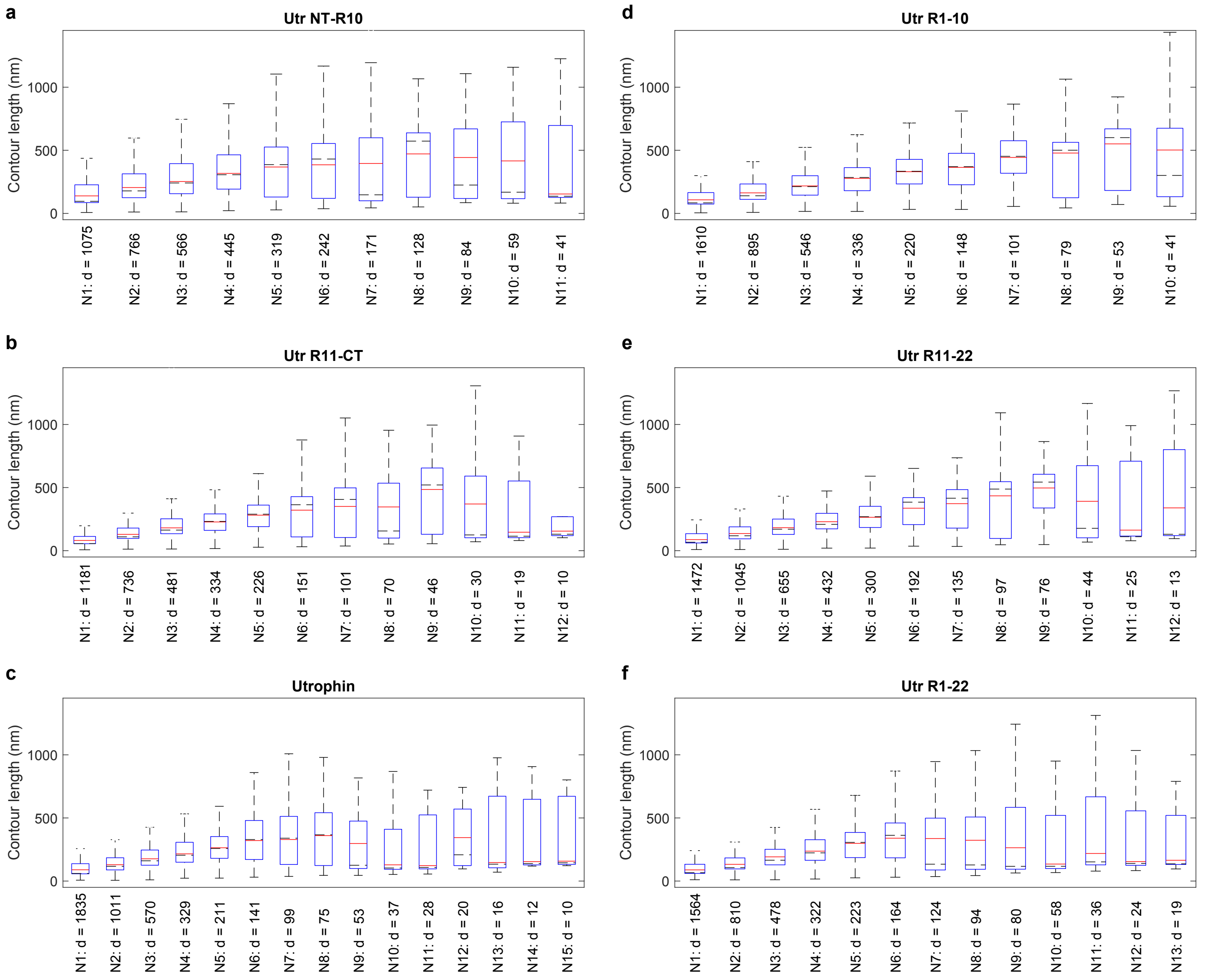

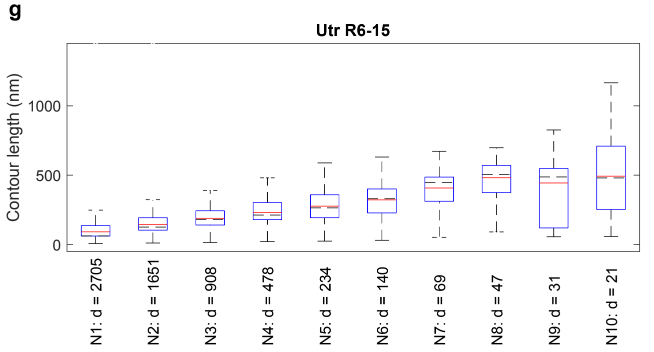

Supplementary Figure S6. Box plots of contour lengths as a function of unfolding count. Box plots of the contour length classified based on the unfolding count, with the red line indicating the median value and the dotted black line indicating the mode. The edges of the box represent the 25% and 75% percentiles, with the whisker plots marking the minimum and maximum recorded values excluding the outliers. These figures show contour length increasing linearly with unfolding events, where unfolding events are represented as ‘Ni’, i = {1,2,3…} and ‘d’ denotes the number of pulling experiments that resulted in ‘Ni’ events. A nearly linear increase in the contour length is expected as the unfolding events occur.

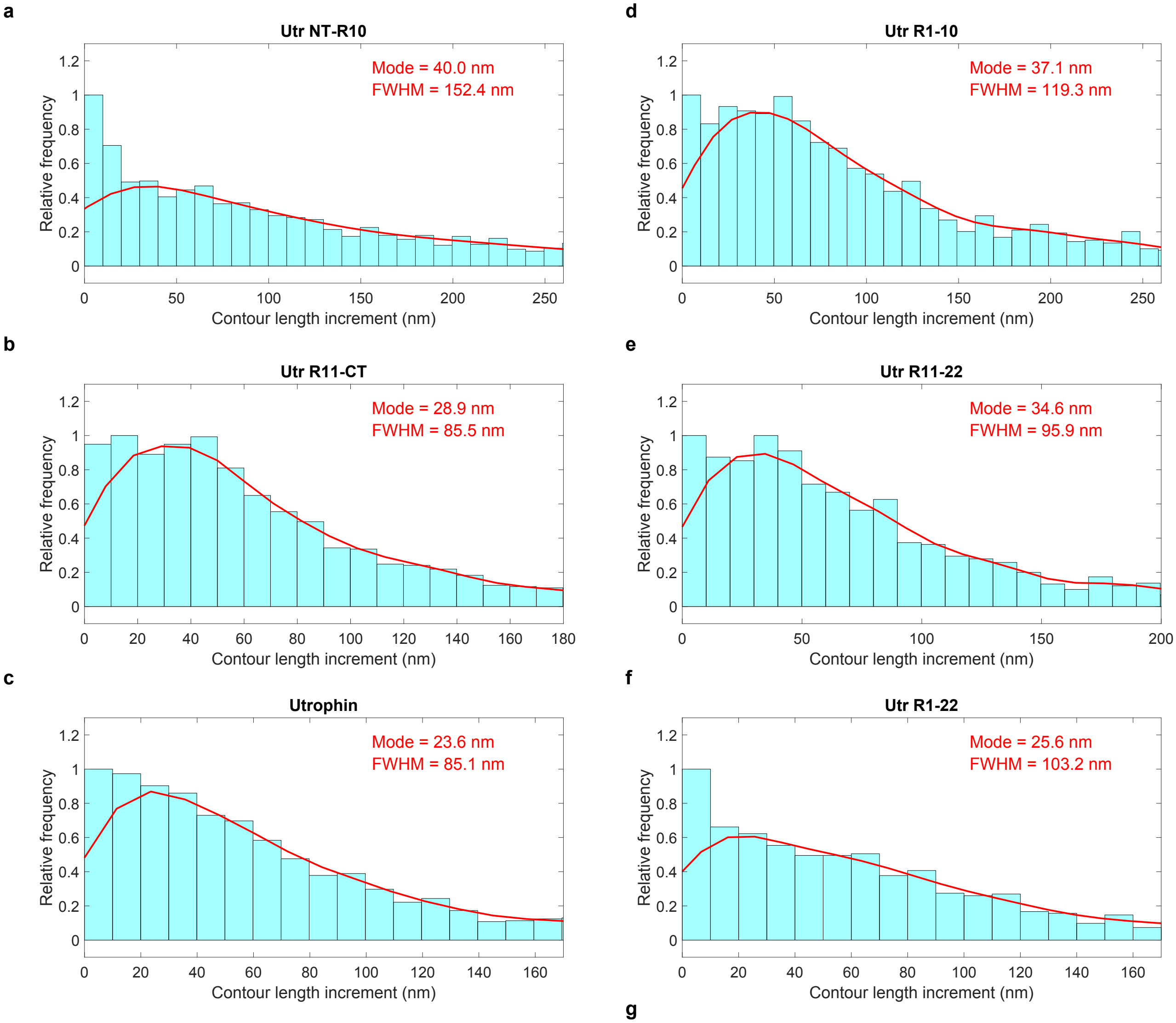

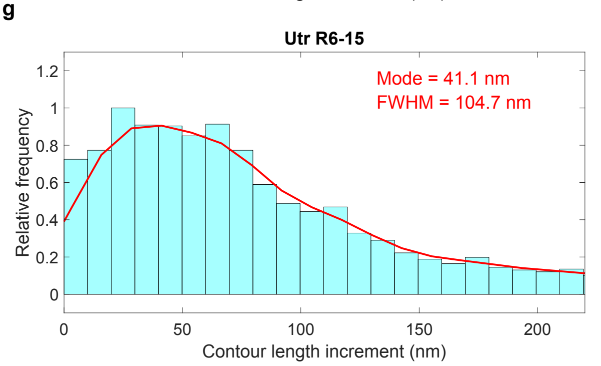

Supplementary Figure S7. Contour length increment histograms for the seven utrophin constructs. The most probable contour length increments range between 23.6 to 41.1 nm. In previous reports, spectrin repeats of 106 amino acids had an average contour length increment of 31.7 nm[15] which gives a predicted contour length increment of 32.6 nm for utrophin repeats of 109 average amino acids.

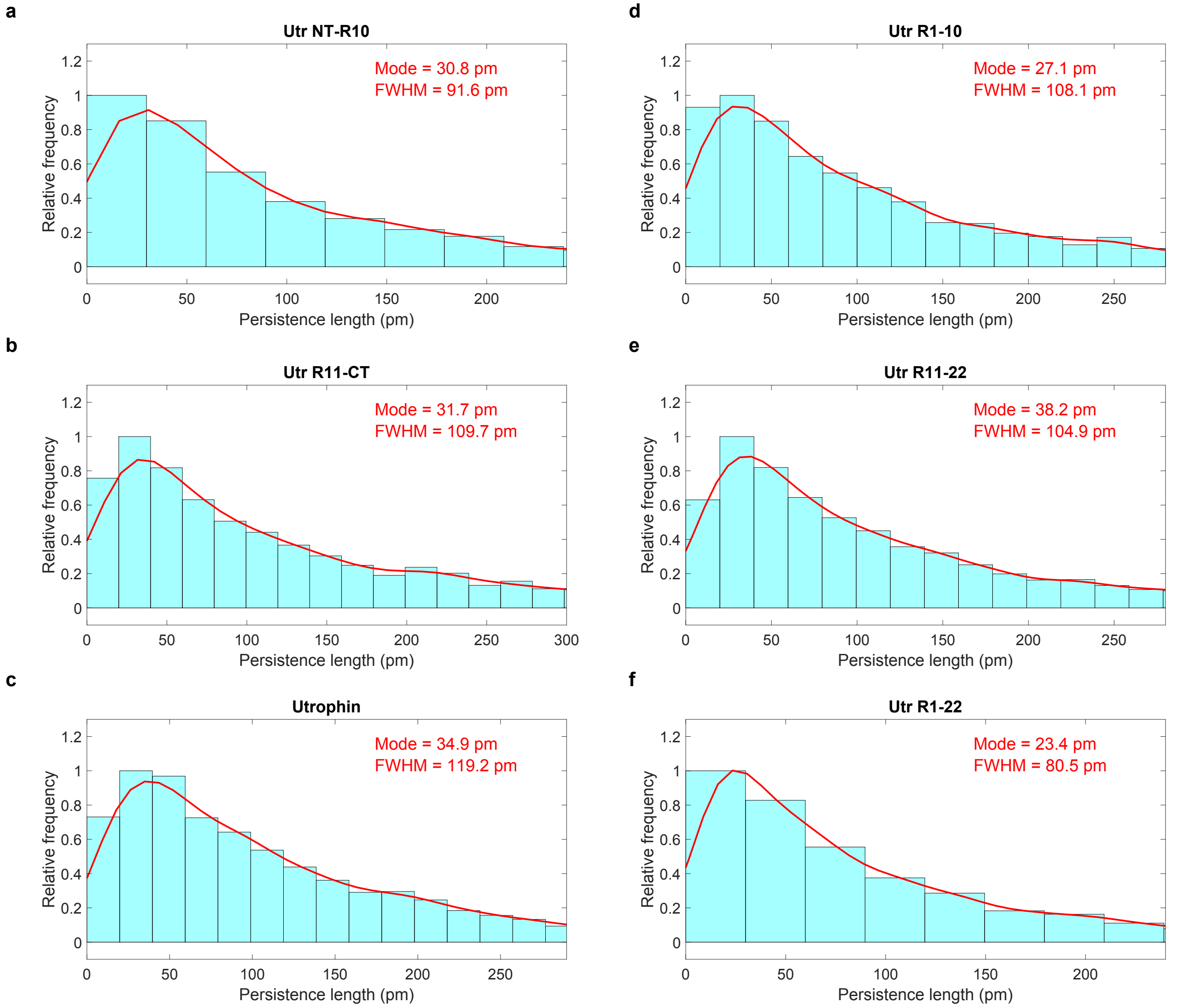

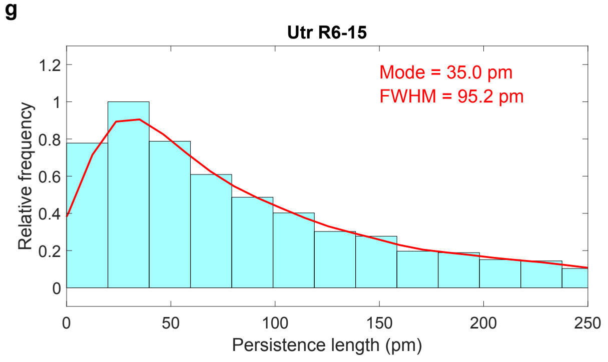

Supplementary Figure S8. Persistence length histograms for the seven utrophin constructs. The most probable persistence lengths were found to be between 23.4 to 38.2 pm for the utrophin constructs.

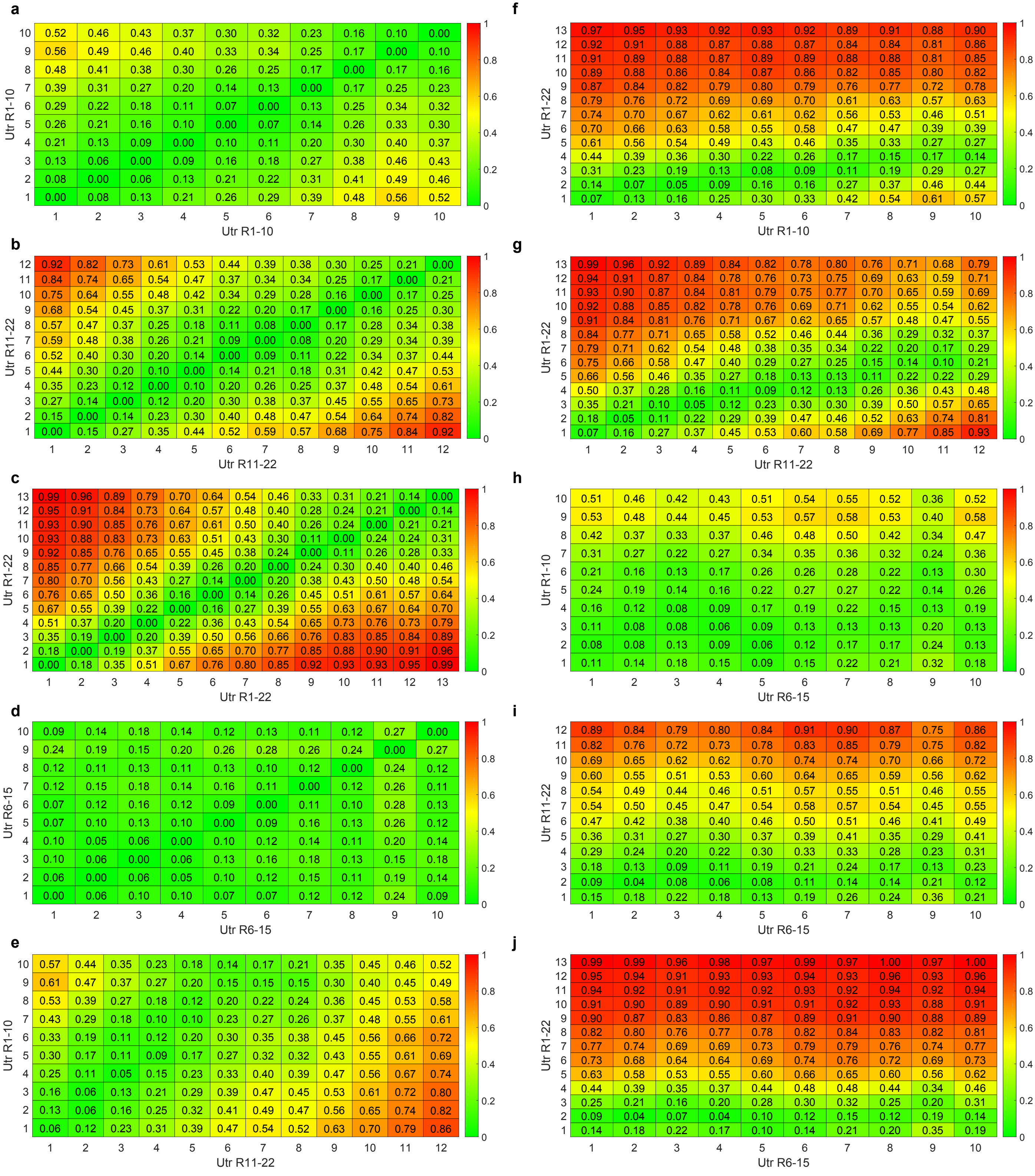

Supplementary Figure S9. Heat maps of Kolmogorov-Smirnov (KS) statistics. KS test metrics which compare the distribution of unfolding forces for different unfolding event counts. The color-bars (heat maps) represent the KS metric with a 0 value (green) indicating similarity and value of 1 representing maximum dissimilarity (red).

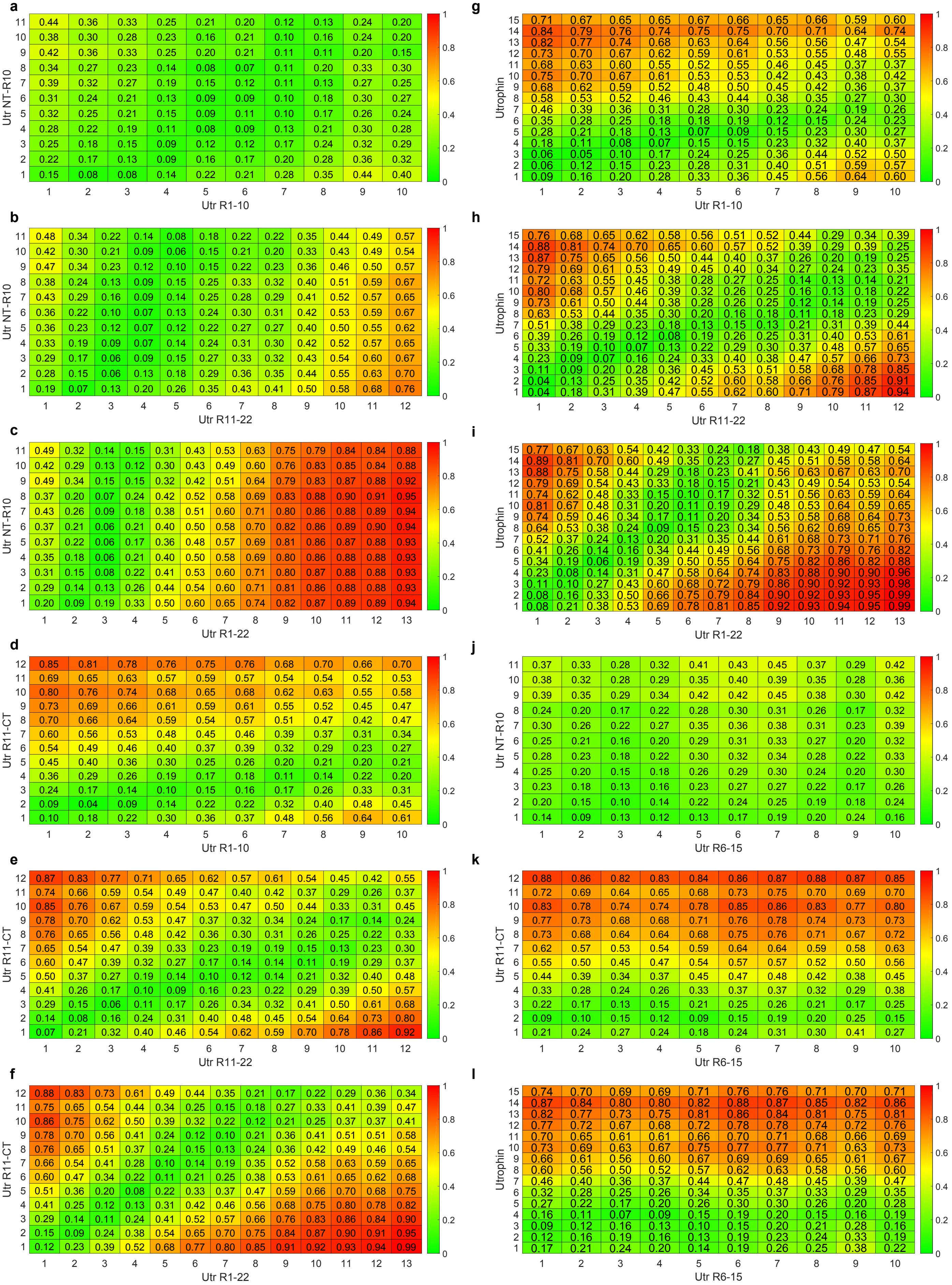

Supplementary Figure S10. Heat maps of Kolmogorov-Smirnov (KS) statistics. KS test metrics which compare the distribution of unfolding forces for different unfolding event counts. The color-bars (heat maps) represent the KS metric with a 0 value (green) indicating similarity and value of 1 representing maximum dissimilarity (red).

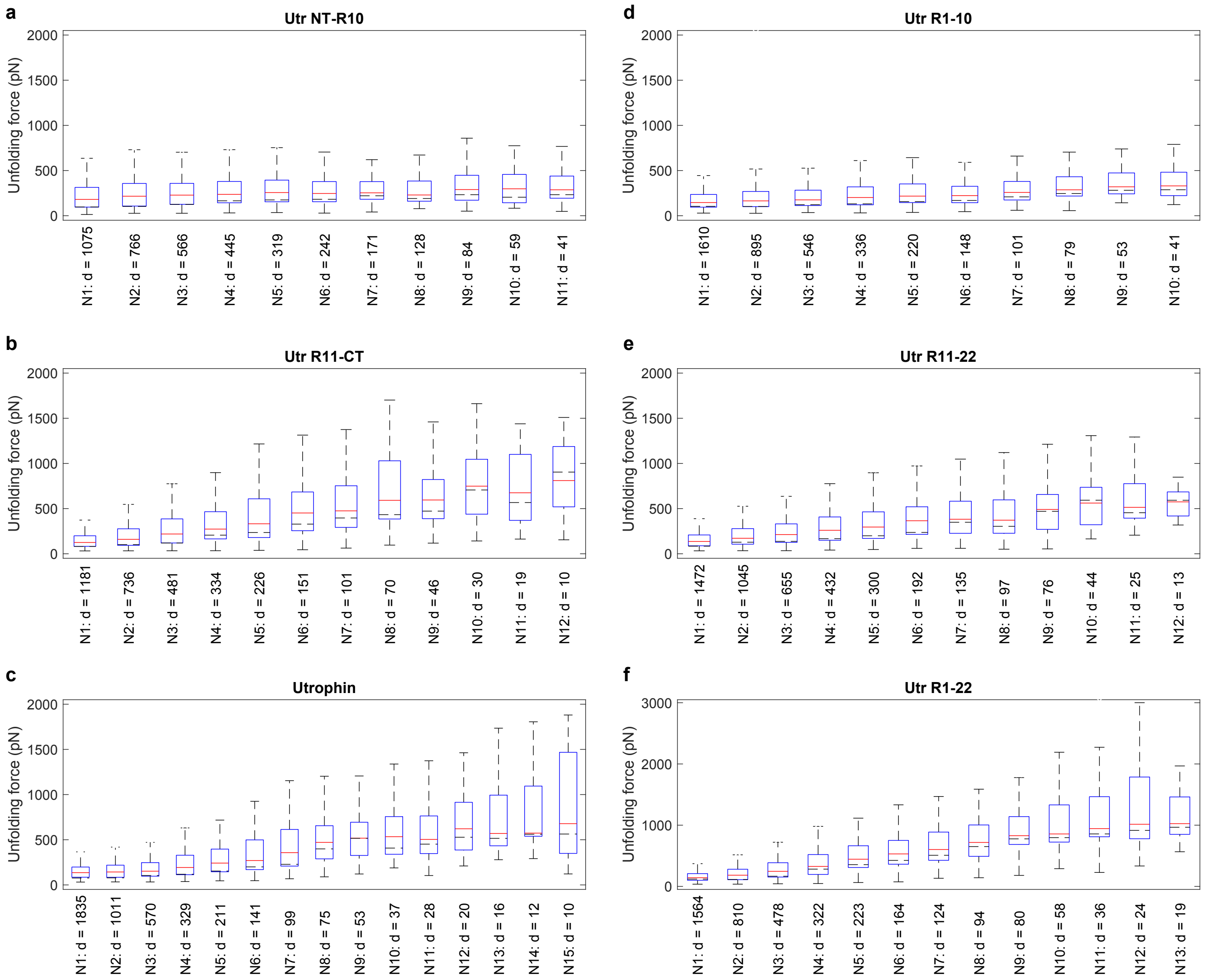

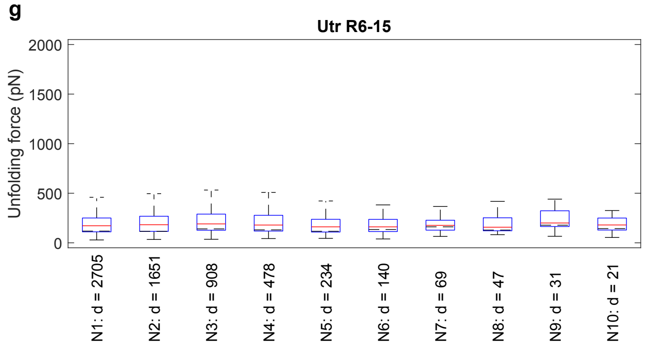

Supplemental Figure S11. Box plots of unfolding forces as a function of unfolding count. Box plots of the unfolding forces classified based on the unfolding count, with the red line indicating the median value and the dotted black line indicating the mode. The edges of the box represent the 25% and 75% percentiles, with the whisker plots marking the minimum and maximum recorded values excluding the outliers. Unfolding events are represented as 'Ni', i = {1,2,3…} and 'd' denotes the number of pulling experiments that resulted in 'Ni' events. Utr NT-R10, Utr R1-R10, and Utr R6-15 (**a**, **d,** and **g**) can be seen to exhibit brittle behavior, wherein the unfolding force for the 10th unfolding event is comparable to that of the 1st unfolding event. Utr R11-CT and UTR R11-R22 show a stiffening spring behavior, wherein the unfolding forces have a 3 to 5-fold increase for the 10th unfolding event compared to the 1st (**b** and **e**). Full-length utrophin and Utr R1-22 show a strongly stiffening spring behavior with 4 to 6-fold increases for the 10th unfolding events compared to their 1st (**c** and **f**). The relative frequencies of each unfolding event are represented in Supplemental Figure S13.

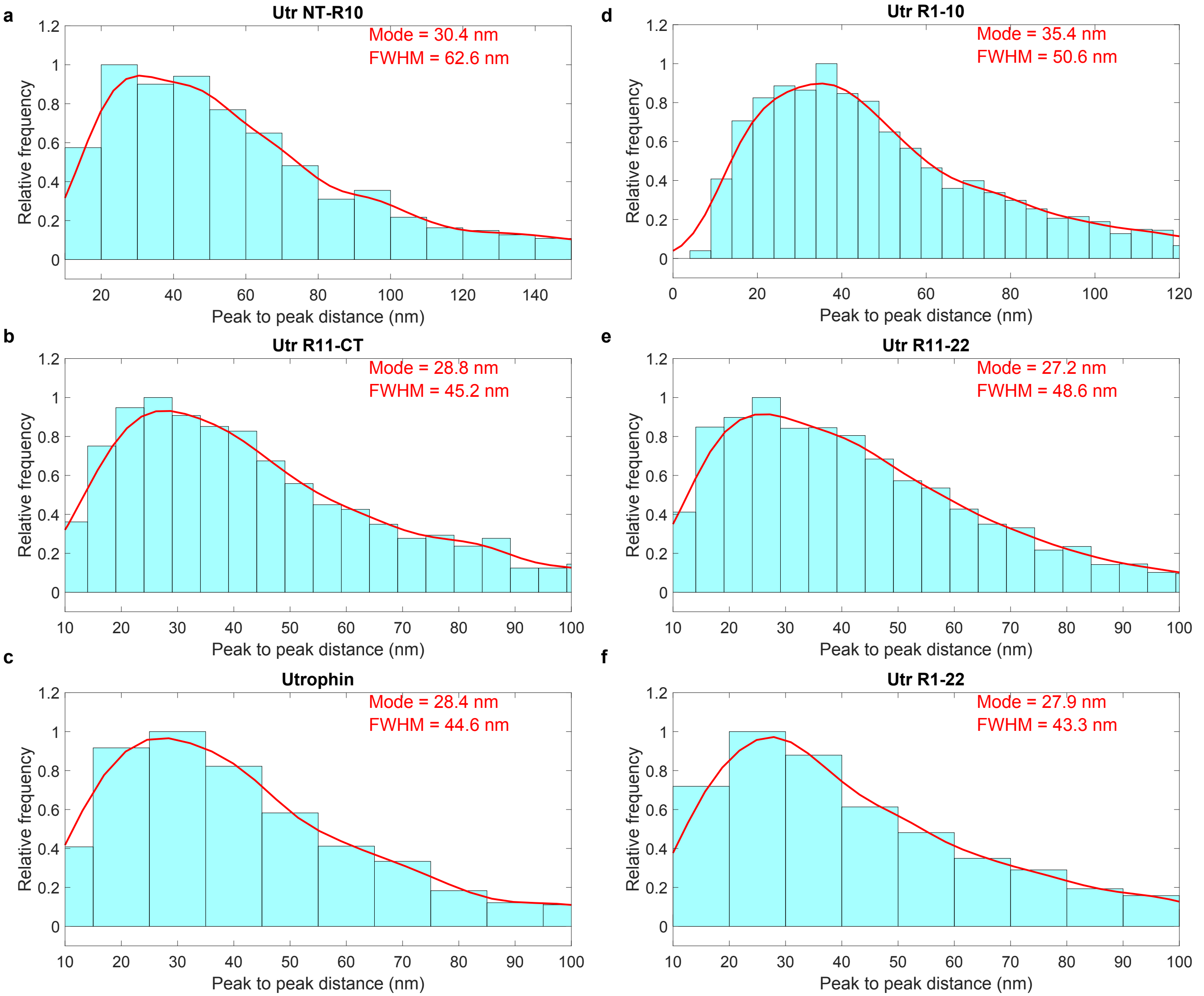

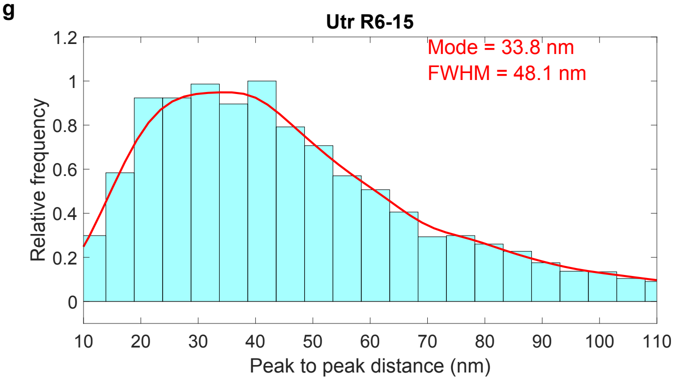

Supplementary Figure S12. Peak to peak distance. Distribution of the elongation of the utrophin constructs in between successive unfolding events. Each construct exhibits an asymmetric distribution with single peak (mode).

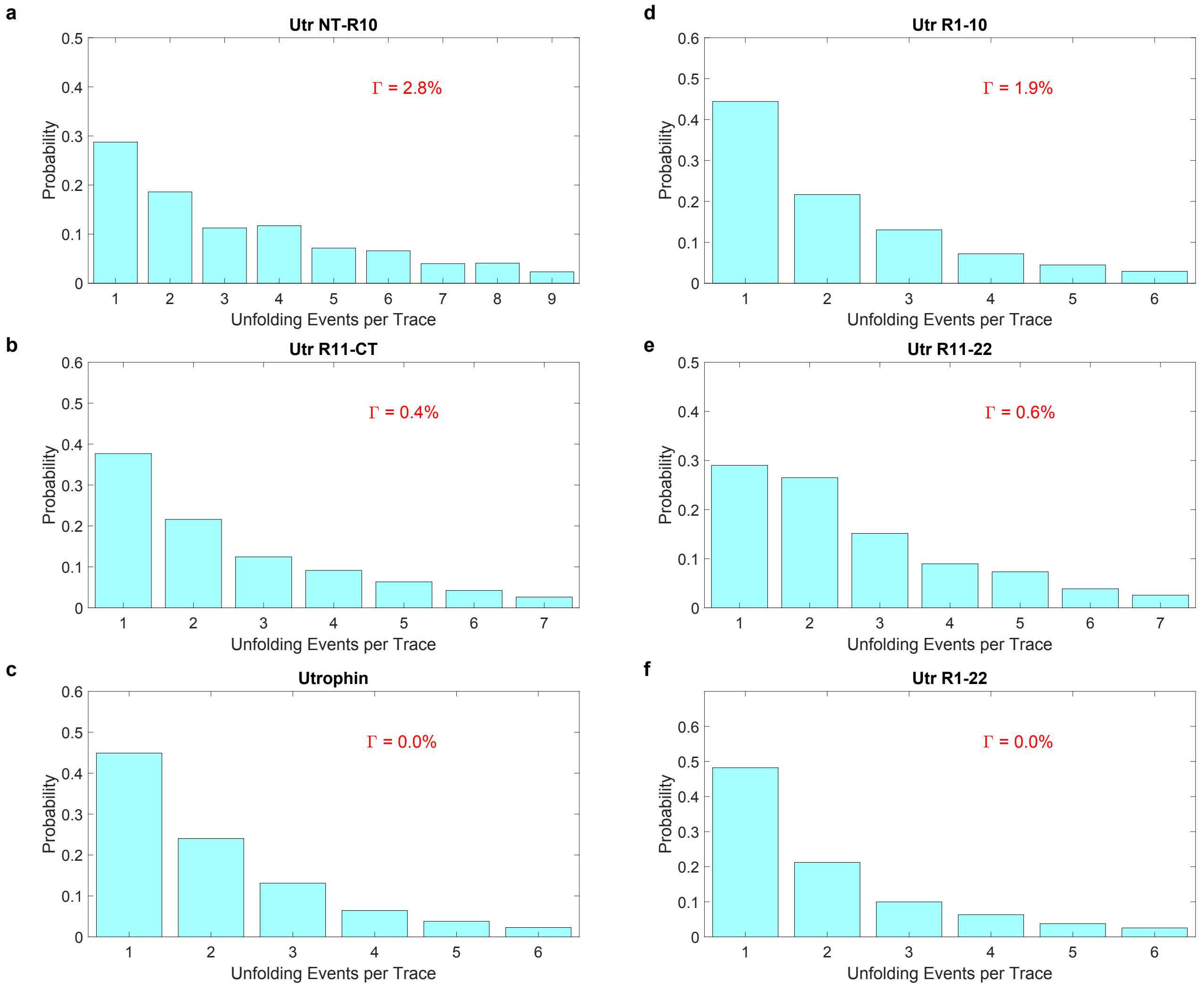

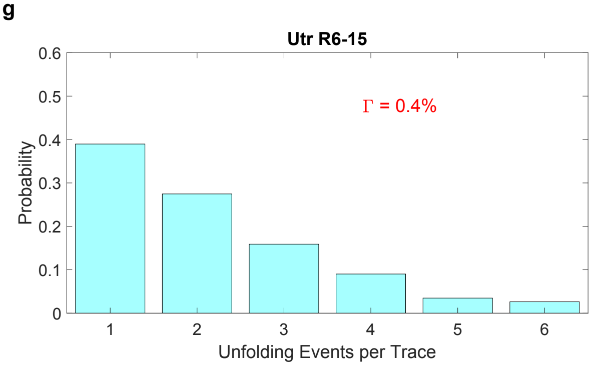

Supplementary Figure S13. Unfolding events per trace. The probabilities of observing a particular number of events (vertical axes) consistently reduce with the increasing unfolding event counts (horizontal axes). The value Γ inset in the figures represents the probability of observing force vs extension curves where the total number of unfolding events exceeds the expected number of folded domains for the given protein construct. The highest value of Γ observed is 2.8% (**a**).

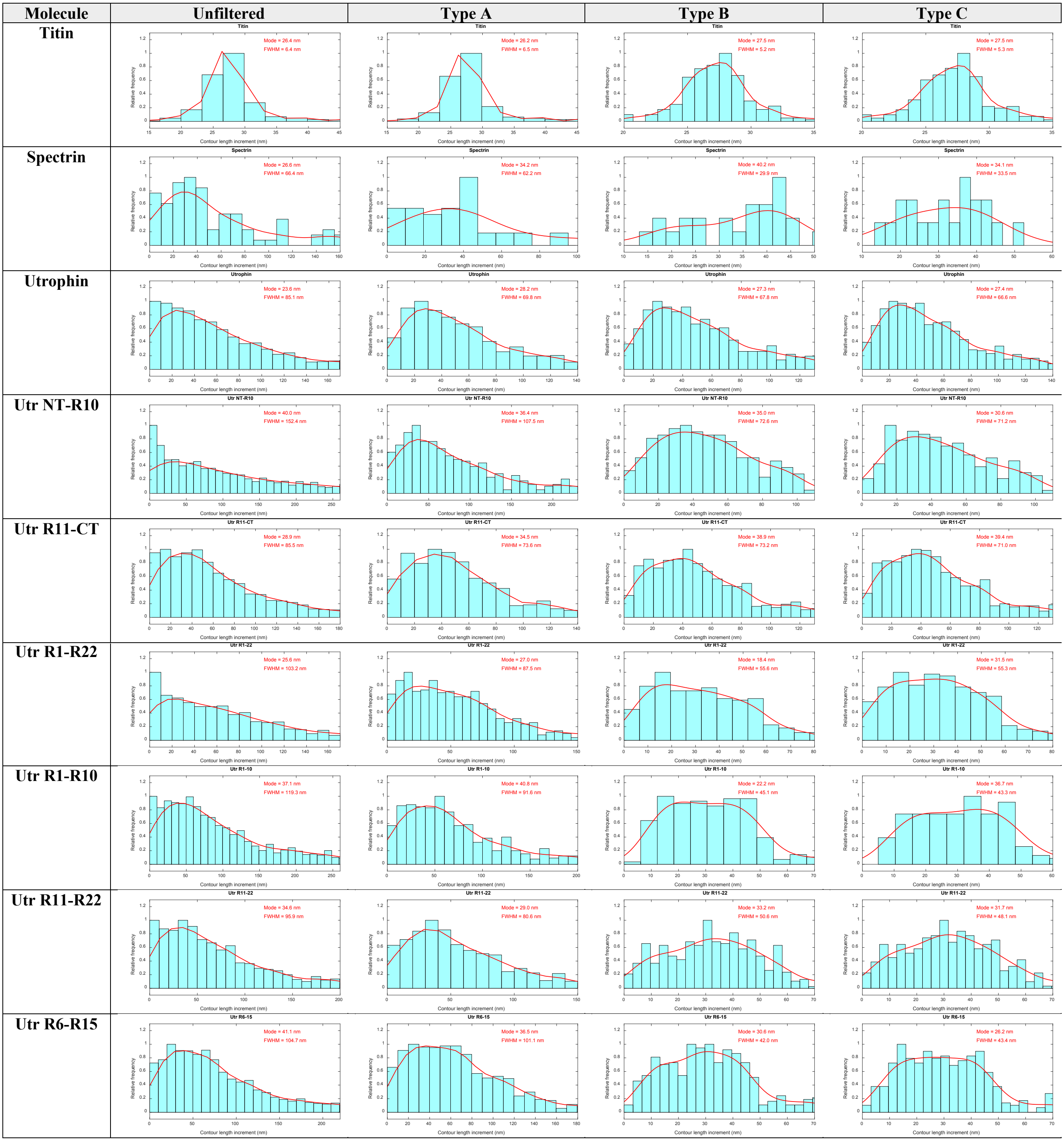

Supplementary Figure S14. Filtered contour lengths. Contour length increment distributions for the reference and utrophin constructs with varying levels of filtering (described in methods).

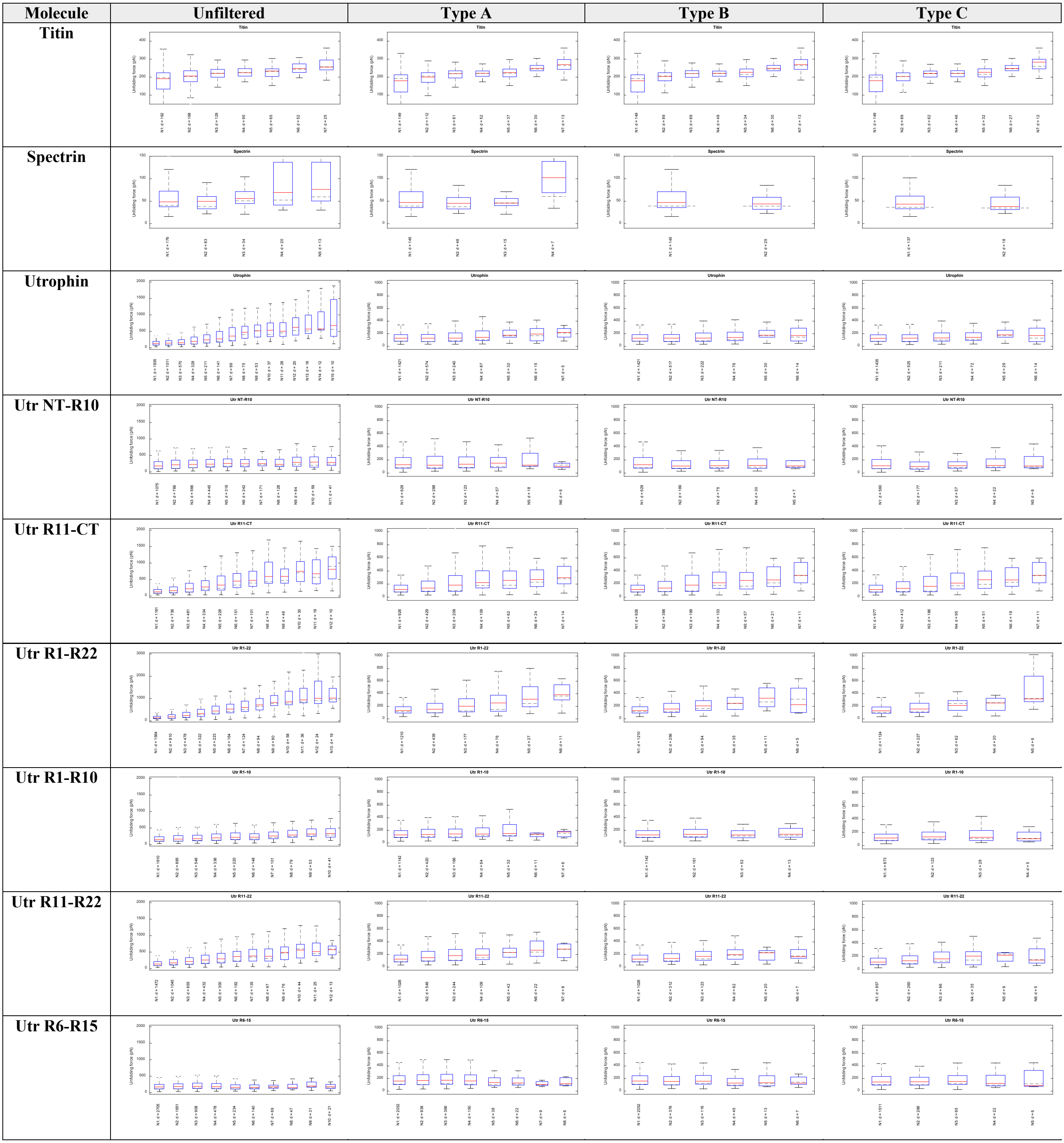

Supplementary Figure S15. Filtered Unfolding Force vs. Event Count Data. Unfolding force trends for the different reference and utrophin constructs with varying levels of filtering (described in methods).

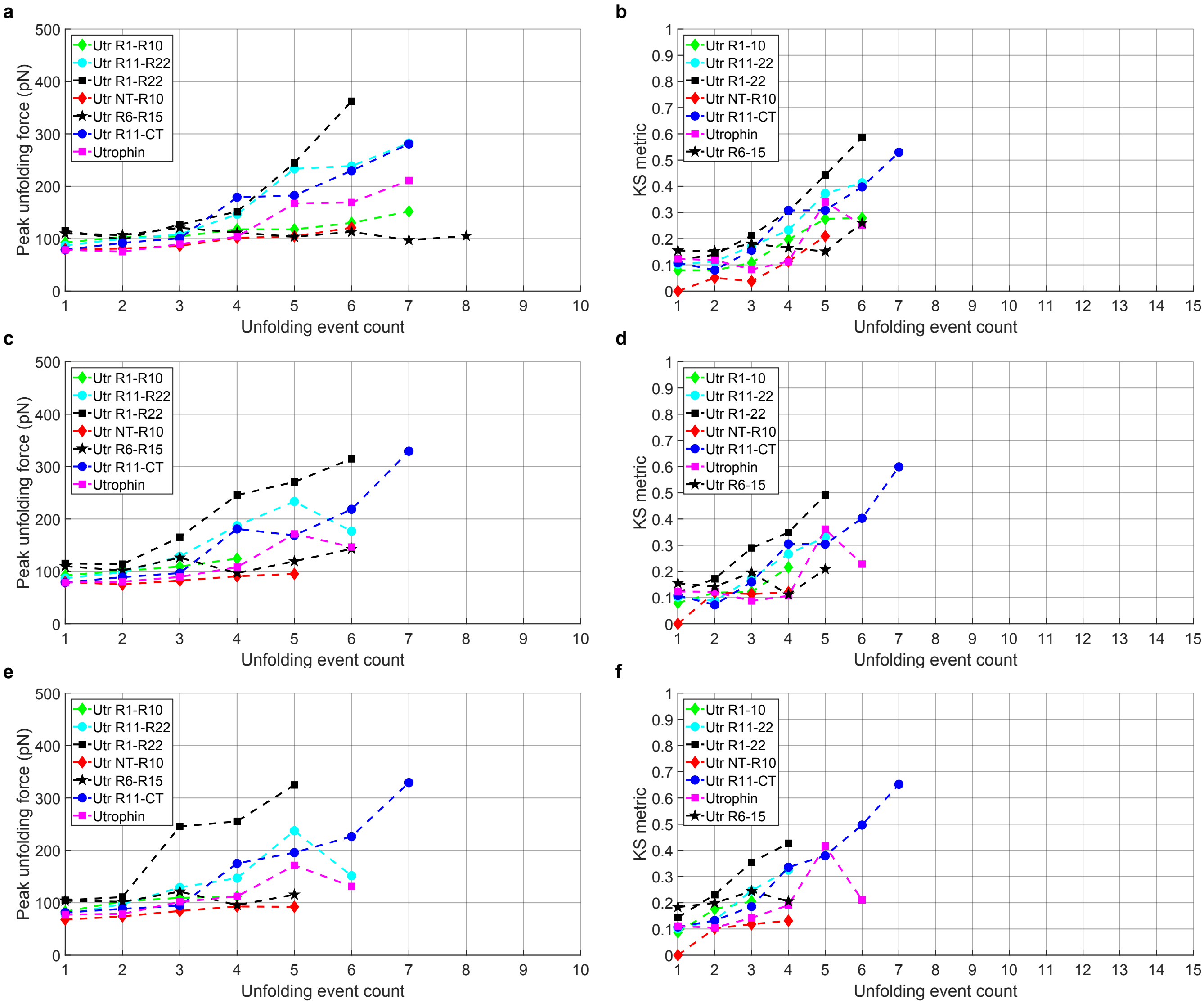

Supplementary Figure S16. Filtered Peak Unfolding Forces and KS metrics. Comparison of peaks (or modes) of unfolding force distribution vs unfolding event counts (**a**, **c**, and **e**) and KS metrics (**b**, **d**, and **f**) with Type A (**a-b**), Type B (**c-d**), or Type C (**e-f**) filtering (described in methods). In comparison to Figure 5, the KS metrics indicate the presence of distinct mechanical behaviors of the N- and C-terminal halves of utrophin despite filtering.

Supplementary Table S1. *Ex vivo* physiology parameters for EDL and soleus muscles.

	EDL		Soleus	
	**utrn +/+**	**utrn -/-**	**utrn +/+**	**utrn -/-**
**EDL or soleus mass (mg)**	10.8 ± 0.3	11.0 ± 0.2	8.5 ± 0.5	8.3 ± 0.3
**L_0 (mm)**	12.8 ± 0.1	13.1 ± 0.1*	12.5 ± 0.1	12.9 ± 0.2
**CSA (cm^2)**	0.018 ± 0.0004	0.018 ± 0.0003	0.010 ± 0.0005	0.009 ± 0.003
**passive stiffness (N/m)**	10.4 ± 0.3	9.6 ± 0.2*	11.4 ± 0.2	10.7 ± 0.2*
**active stiffness (N/m)**	862.0 ± 22.3	830.4 ± 19.4	510.1 ± 36.8	478.2 ± 13.8
**P_0 (mN)**	412.6 ± 7.6	428.2 ± 8.7	208.4 ± 10.0	217.0 ± 6.0
**specific P_0 (N/cm^2)**	23.4 ± 0.6	23.8 ± 0.3	23.3 ± 0.8	25.5 ± 0.8

Physiology parameters for wild-type (utrn +/+) and utrophin null (utrn -/-) mice with mean ± standard error of the mean. EDL, extensor digitorum longus; L_0, muscle length; CSA, physiological cross sectional area, P_0, peak isometric tetanic force. For measurements of passive stiffness in soleus and EDL muscle as well as L_0 EDL of utrn -/- mice, *$p<0.05$ compared to utrn +/+. n=11 for utrn +/+ and n=14 for utrn -/-.
